# A Computational Model Integrating Multiple Phenomena on Cued Fear Conditioning, Extinction, and Reinstatement

**DOI:** 10.3389/fnsys.2020.569108

**Published:** 2020-09-29

**Authors:** Andrea Mattera, Marco Pagani, Gianluca Baldassarre

**Affiliations:** Institute of Cognitive Sciences and Technologies, National Research Council, Rome, Italy

**Keywords:** amygdala, prefrontal cortex, endocannabinoids, fear conditioning, fear extinction

## Abstract

Conditioning, extinction, and reinstatement are fundamental learning processes of animal adaptation, also strongly involved in human pathologies such as post-traumatic stress disorder, anxiety, depression, and dependencies. Cued fear conditioning, extinction, restatement, and systematic manipulations of the underlying brain amygdala and medial prefrontal cortex, represent key experimental paradigms to study such processes. Numerous empirical studies have revealed several aspects and the neural systems and plasticity underlying them, but at the moment we lack a comprehensive view. Here we propose a computational model based on firing rate leaky units that contributes to such integration by accounting for 25 different experiments on fear conditioning, extinction, and restatement, on the basis of a single neural architecture having a structure and plasticity grounded in known brain biology. This allows the model to furnish three novel contributions to understand these open issues: (a) the functioning of the central and lateral amygdala system supporting conditioning; (b) the role played by the endocannabinoids system in within- and between-session extinction; (c) the formation of three important types of neurons underlying fear processing, namely fear, extinction, and persistent neurons. The model integration of the results on fear conditioning goes substantially beyond what was done in previous models.

## 1. Introduction

Conditioning, extinction, and reinstatement are learning processes that play a fundamental role in animal adaptation (Maren, 2005). These processes rely on the amygdala, an ancient brain nucleus playing a key role in emotional regulation, and on cortical areas such as the medial prefrontal cortex (mPFC), very important for the regulation of lower brain centers (Vidal-Gonzalez et al., 2006; Mirolli et al., 2010; Pape and Pare, 2010; Johansen et al., 2011; Sierra-Mercado et al., 2011; Giustino and Maren, 2015). The dysfunction of these systems is also strongly involved in important human pathologies (post-traumatic stress disorder, anxiety, depression, and dependencies; Davidson, 2002; Koenigs and Grafman, 2009a,b; Peters et al., 2009; Likhtik et al., 2014).

Cued fear conditioning is one of the most important experimental paradigms allowing the study of conditioning, extinction, and restatement. The procedure for *conditioning* consists in three/four pairings of a neutral stimulus (conditioned stimulus, CS), usually an auditory tone, with an electric shock (unconditioned stimulus, US). After conditioning, animals responds to a CS presentation with behavioral manifestations of fear, like freezing. If, after conditioning, the CS is delivered many times without the US, fear behavior undergoes a gradual *extinction*. *Reinstatement* involves different experimental protocols for the re-establishment of the CS-induced freezing. For example, after the simple exposure to the US, the fear behavior is manifested again (Maren and Holmes, 2016).

A rich experimental literature on the neural substrates underlying cued fear conditioning, extinction, and reinstatement revealed a very complex picture, involving many brain areas, such as the amygdala and the mPFC, and both long term potentiation (LTP) and depression (LTD) (Herry et al., 2010; Janak and Tye, 2015). Regarding conditioning, this causes LTP within the pathway conveying the conditioned stimulus (CS) from the auditory thalamus and cortex to the lateral nucleus of the amygdala (LA), here referred to as “CS-pathway” (Rogan and LeDoux, 1995; McKernan and Shinnick-Gallagher, 1997; Rogan et al., 1997; Tsvetkov et al., 2002). Optogenetic depotentiation of the CS-pathway (Nabavi et al., 2014; Kim and Cho, 2017), or the application of inhibitors of LTP consolidation in LA (Schafe et al., 2005), abolishes conditioning, demonstrating that LTP taking place in this pathway is necessary for establishing fear memories. On the other hand, artificial induction of LTP within the CS-pathway is not sufficient to obtain conditioning (Nabavi et al., 2014), implying that fear conditioning involves more complex plasticity phenomena. In particular, another key area important for conditioning is the lateral subdivision of the central amygdala (CeL): its inhibition by GABA agonists impairs the US-CS association (Wilensky et al., 2006; Ciocchi et al., 2010). However, how the US and CS reach CeL is unknown. CeL receive afferents from LA, but not from the auditory thalamus, directed to a population of interneurons that strengthen their activation with conditioning (Li et al., 2013). Even though the US could reach CeL through a direct connection from the parabranchial nucleus (Han et al., 2015), it has been shown that conditioning can be obtained pairing an auditory tone and the optogenetic stimulation of LA, which replaces the US (Johansen et al., 2010). This suggests that US information sufficient for conditioning reach CeL through a relay in LA. However, at the moment the specific circuits and the plasticity mechanisms supporting the involvement of CeL in fear conditioning are not fully understood.

Another region important for fear memory is the mPFC, that comprises the infralimbic (IL) and the prelimbic cortex (PL), and is reciprocally connected to the basal nucleus of the amygdala (BA). Interestingly, the mPFC is necessary for the expression of learned fear but not for its formation (Corcoran and Quirk, 2007); moreover, it has been shown (Vouimba and Maroun, 2011) that conditioning induces the LTP of the connection between the mPFC and the neurons in the BA.

Conditioning establishes a fear circuit comprising two different groups of neurons, the so-called *fear neurons*, located in BA (Herry et al., 2008) and in the medial subdivision of the central amygdala (CeM; Amano et al., 2010), and the *persistent neurons*, located in LA (Repa et al., 2001; An et al., 2012; Feng et al., 2016) and BA (Amano et al., 2011; Trouche et al., 2013). After fear conditioning has been established, both fear and persistent neurons fire in response to the CS. However, the CS-related response of fear neurons fades away after fear extinction, while persistent neurons continue to be activated by the CS even without behavioral responses. This means that a trace of the fear memory remains in the amygdala and extinction does not lead it back to the pre-conditioning state. In fact, extinction traces can be reverted: the simple administration of the US is capable of reinstating the fear response to the CS (Maren and Holmes, 2016).

Extinction induces both LTP and LTD at some key synapses involving the IL, BA and the intercalated cells of the amygdala (ITC). In particular, synaptic connections from BA to the mPFC and from BA to the ITC are potentiated, while those from the mPFC to BA are depotentiated (Amano et al., 2010; Vouimba and Maroun, 2011; Cho et al., 2013). Interestingly, the CS-pathway established by LTP during conditioning is not eliminated by fear extinction (Clem and Huganir, 2010; Kim and Cho, 2017). Reinstatement is associated with changes in the opposite direction: the connections from BA to the mPFC are depotentiated while the connections from the mPFC to BA are potentiated (Vouimba and Maroun, 2011).

As a result of the processes of plasticity, extinction causes the emergence of a third class of CS-responsive neurons, the so-called *extinction neurons*, located in BA (Herry et al., 2008), in ITC (Amano et al., 2010), and in the IL (Milad and Quirk, 2002; Santini et al., 2008). The inhibiting of BA fear neurons by interneurons represent a key mechanism of extinction (Chhatwal et al., 2005; Pape and Pare, 2010; Trouche et al., 2013; Asede et al., 2015). In particular, the ITC interneurons play a central role in the process because their damage produces a deficit in the extinction recall (Likhtik et al., 2008). A similar deficit occurs with the lesion of the IL neurons (Quirk et al., 2000; Burgos-Robles et al., 2007; Laurent and Westbrook, 2009; Bloodgood et al., 2018).

It has been shown that the extinction actually involves a short-lasting within-session extinction, and a long-lasting between-session extinction. While lesions or pharmacological impairment of the IL or the ITC do not prevent the within-session extinction, they abolish the between-session extinction. In particular, during a session the freezing progressively reduces with the repeated presentation of the CS, but the day after it returns to pre-extinction levels (Quirk et al., 2000; Burgos-Robles et al., 2007; Likhtik et al., 2008; Laurent and Westbrook, 2009; Do-Monte et al., 2015; Bloodgood et al., 2018).

Besides the activation of the IL and the ITC, one of the most important factors regulating extinction is the presence of endocannabinoids. Indeed, an impairment of the endocannabinoid system compromises both the within- and the between-session extinction (Marsicano et al., 2002). Endocannabinoids are produced by some BA and CeM postsynaptic neurons after prolonged depolarization and diffuse to the presynaptic terminal, where they bind the receptor CB1 and induce a transient reduction of neurotransmitter release. This effect is called *Depolarization-induced suppression of Inhibition/Excitation* (DSI/E) if the presynaptic target is, respectively a GABAergic/glutamatergic neuron (Kano et al., 2009; Kamprath et al., 2011). Experiments show that a local impairment of endocannabinoids in CeM blocks only the within-session extinction, whereas an impairment in BA's endocannabinoids affects the between-session, but not the within-session, extinction (Kamprath et al., 2011). This suggests that the endocannabinoids system is activated transiently in BA to produce between-session extinction, but the specific way this happens is still unknown.

The aim of this work is to present a comprehensive computational model, based on firing rate leaky units, that relies on those experimental findings and proposes specific hypotheses to overcome the knowledge gaps described above. The model is biologically grounded, in particular it is formed by connections corresponding to relevant pathways of the brain drawn from the literature on fear conditioning. The functioning and the learning processes of the model have been validated through the simulation of 25 different experimental findings in a coherent fashion.

## 2. Materials and Methods

### 2.1. Overview of the Model

**Figure 1:**
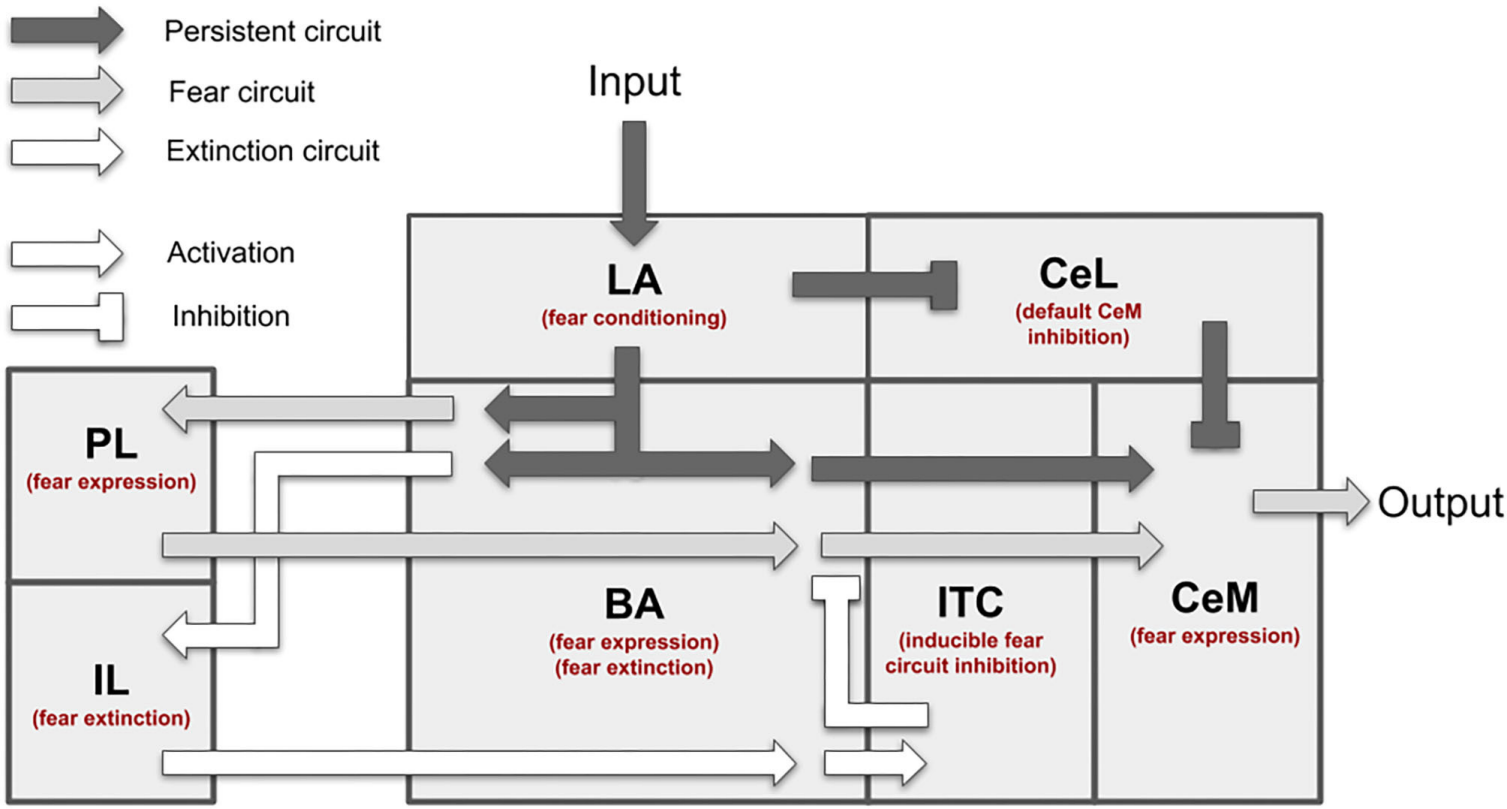
Overview of the brain areas reproduced by the model, and their main connections and functions played in conditioning and extinction. The scheme shows how the input (CS, US) is conveyed from LA to CeM through three main circuits: a persistent circuits (LA-BA-CeM and LA-CeL-CeM), a fear circuit (LA-BA-PL-BA-CeM), and an extinction circuit (BA-IL-BA-ITC-BA-CeM), within which the persistent/fear/extinction neurons are found.

Figure 1 presents an overview of the brain areas reproduced in the model, the main information flows exchanged by them, and the possible overall role they play. The input of the model (the CS and the US) reaches LA (Romanski et al., 1993; Blair et al., 2001; Wolff et al., 2014; Krabbe et al., 2018; Rhomberg et al., 2018), while the output (freezing) is expressed by CeM (Pape and Pare, 2010). LA receives from the somatosensory and the auditory thalamus and cortex and projects to CeL and BA (Stefanacci et al., 1992; Pitkänen et al., 1995; Savander et al., 1997; Pape and Pare, 2010; Li et al., 2013). The main function of CeL is to operate as a tonic “brake” on the CeM activity. When LA principal neurons are activated, they exert an inhibition on the output neurons of CeL, removing the brake on CeM (Li et al., 2013). Even in the absence of the brake, CeM is not active without an input from BA. BA receives afferent connections from LA and projects to the PL, the IL, and CeM (Courtin et al., 2014; Senn et al., 2014; Asede et al., 2015; McGarry and Carter, 2016; Cummings and Clem, 2020). The PL and IL, in turn, project back to BA (Vertes, 2004; Cho et al., 2013; Courtin et al., 2014). The PL is important for fear expression, while the IL drives extinction (Quirk et al., 2000; Vidal-Gonzalez et al., 2006; Corcoran and Quirk, 2007). The part of BA that receives connections from the PL projects to CeM to express freezing. Information passing through the IL, instead, projects to the ITC and then back to BA to inhibit freezing expression (Vertes, 2004; Pinard et al., 2012; Cho et al., 2013; Asede et al., 2015). The BA-CeM circuit inhibited by the ITC is the part of the conditioning circuit that is reversed by extinction, while the remaining part is persistent. Overall, this system leads the CS and US input to reach the CeM output through three main circuits: a “persistent circuits” (LA-BA-CeM and LA-CeL-CeM), a “fear circuit” (LA-BA-PL-BA-CeM), and an “extinction circuit” (BA-IL-BA-ITC-BA-CeM), within which persistent, fear, and extinction neurons are, respectively found (Repa et al., 2001; Milad and Quirk, 2002; Herry et al., 2008; Santini et al., 2008; Amano et al., 2010, 2011; An et al., 2012; Trouche et al., 2013).

### 2.2. Neural Units of the Model

The neural units forming the model are *leaky units* (Dayan and Abbott, 2001) simulating the main dynamical properties of neurons through differential equations. In particular, each unit represents a population of cells with the same biological properties. The decision about the populations to insert in the model was done on the basis of the different neuronal types (pyramidal, parvalbumin, somatostatin), the relevant neuronal regions considered (e.g., the pyramidal neurons in the mPFC and the pyramidal neurons in the LA) and the different connectivity (e.g., pyramidal neurons in the BA that project to PL and pyramidal neurons in the BA that project to IL etc).

Following rate-based leaky-neuron models we abstracted over the electrophysiological details of neurons (e.g., John et al., 2013; Carrere and Alexandre, 2015; Oliva et al., 2018). However, instead of using completely ungrounded parameters, we tried to capture some of the differences between the neuronal populations used in the model, for example the relative sizes of the maximum firing rate of populations of parvalbumin, pyramidal, or somatostatin neurons. Analogously, in choosing the tau of the units, that are usually set arbitrarily in firing rate models, in the absence of information about the population time constants we set them in proportion to those of the corresponding single neurons.

The equations of the neuron dynamics were approximated with the Euler method to implement them in discrete time steps (dt = 10 ms) as requested by computer simulations. The dt used in the simulations are rather high with respect to the used τ coefficients. This was done to speed up the simulations, in particular those related to the sensitivity analysis that would have been otherwise computationally infeasible. To be sure that the high dt would not make the model unstable or give different results, we confronted the simulation in **Figure 3** (on the key target phenomena) run with a dt of 1 ms with the one run with a dt of 10 ms. Having observed very similar results in the two conditions (data not shown; the model is very stable as leaky neurons are low-pass filters), we used dt = 10 ms in all the simulations shown in **Figures 3**–**12**.

The change of the membrane potential of a unit was regulated with the following differential equation:

(1) $$\begin{array}{r} \tau \cdot \dot{V}=-V+T+I \end{array}$$

where *V* is the membrane potential, $\dot{V}$ is its first derivative in time, *T* is the tonic activity of the neuron, and *I* is the presynaptic input. τ is the time constant of the unit, having different values for different units. Given that the units of the model represent populations, the setting of the τ parameters should reflect the population temporal dynamics. However, to our knowledge these are not known for amygdala. Generally for firing rate leaky models of the amygdala the setting of such parameters is done with arbitrarily (e.g., 0.001 in John et al., 2013; 0.005 in Oliva et al., 2018; 0.05 in Carrere and Alexandre, 2015). Given this uncertainty, rather than making a fully arbitrary choice we set the values to those of the single real neurons of the amygdala and the mPFC: this had the important advantage of allowing us to at least reflect the *relative* size of the parameters of the different populations considered. For the overall scale, we decided to leave it to the values of the single neurons (from 7.7 to 35.6 ms, see Supplementary Table 1).

As commonly done, the membrane potential *V* was remapped from the usual values of (−70, +30) mV to a value *U* ranging in (0, *max*) as leaky neurons tend to spontaneously converge to zero (*max* was set equal to π to have a firing rate mostly ranging in the non-saturating part of the transfer function of the neuron discussed below). The biological neurons considered here have a tonic activity that is 0.3 Hz for pyramidal neurons and 1.3 Hz for most classes of interneurons (Chen et al., 2015). Instead, parvalbuminergic neurons have a significant tonic activity, 5.3 Hz (Chadderton et al., 2009). We thus added a suitable constant T to the input of neurons to reflect such relative basal firings. Although the T variables represent abstract quantities, we set them in proportion to the actual basal firing rates indicated above. *I* was calculated as the sum of all the presynaptic input values *A*_*i*_ from other units each multiplied by the respective connection weights *w*_*i*_:

(2) $$\begin{array}{r} I=\underset{i}{\sum }w_{i}\cdot A_{i} \end{array}$$

The activation *I* of the neural unit (firing rate) was then calculated through the follows transfer function:

(3) $$\begin{array}{r} A=\phi \cdot {\left[tanh\left(U-\psi \right)\right]}^{+} \end{array}$$

where [*x*]^+^ is the positive function ([*x*]^+^ = *x* if 0 ≤ *x*, and [*x*]^+^ = 0 if *x* < 0), *tanh*(x) is the hyperbolic tangent function ($tanh\left(x\right)=\frac{e^{x}-e^{-x}}{e^{x}+e^{-x}}$), ψ is the unit threshold (remapped onto the same range as *U*), and ϕ is the maximum firing rate (the values of ψ and ϕ were taken from the literature; see Supplementary Table 1).

### 2.3. Model Functioning

**Figure 2:**
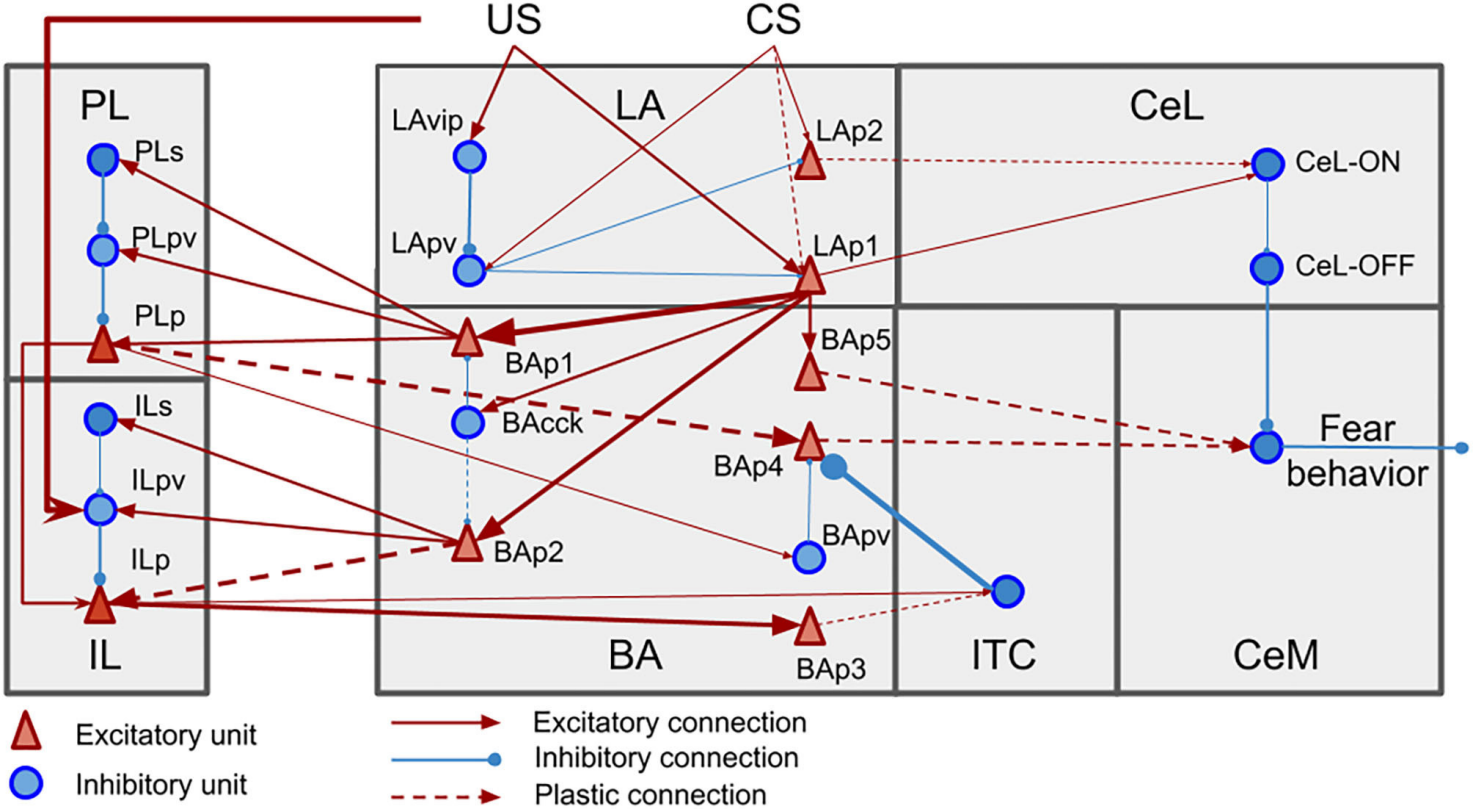
Neural units and connection pathways of the model. The width of the connections is roughly proportional to the connection weights at the beginning of the simulations.

The model is formed by 9 excitatory and 12 inhibitory leaky units (Figure 2). The connections between units are based on the literature, as reported in Supplementary Table 2.

The model main input component is LA, which receives the US and CS from the somatosensory and auditory thalamus and cortex. We implemented CS and US as presynaptic inputs of value 100, multiplied by the weight of the corresponding connection. Pyramidal neurons of LA (LAp1 and LAp2) are negatively regulated by parvalbumin interneurons (LApv) that in turn are inhibited, through a relay (LAvip), by the US (Wolff et al., 2014; Krabbe et al., 2018; Rhomberg et al., 2018). LA reaches CeM, the output component of the model that enacts the conditioned responses (Pape and Pare, 2010), following two pathways: the first through BA units (Stefanacci et al., 1992; Pitkänen et al., 1995; Savander et al., 1997), and the second through two types of interneurons in CeL, called CeL-ON and CeL-OFF (Ciocchi et al., 2010; Haubensak et al., 2010; Li et al., 2013). CeL-OFF neurons have a tonic inhibitory firing that shuts down the activity of CeM. When CeL-ON is activated by LA, it inhibits CeL-OFF, allowing CeM to be depolarized.

BA projects to CeM, the ITC (Amano et al., 2010), and the PL and IL (Senn et al., 2014). Among the pyramidal neurons of the BA (BAp1-5), those projecting to the PL and IL receive the feedforward inhibition of cannabinoid receptor-expressing cholecystokinin-positive basket cells (BAcck), while those receiving afferents from the mPFC receive feedforward inhibition from parvalbumin interneurons BApv (Smith et al., 2000; Cho et al., 2013; Arruda-Carvalho and Clem, 2014; Wolff et al., 2014). The activation of the PL has an excitatory effect on CeM thus having a freezing-promoting effect, while the IL stimulation causes the opposite effect (Vidal-Gonzalez et al., 2006; Amano et al., 2010; Burgos-Robles et al., 2017). The PL and IL receive afferent connections from BA and project back to it (Vertes, 2004; Cho et al., 2013; Courtin et al., 2014; Bloodgood et al., 2018). To do this, the inhibitory control of parvalbumin interneurons of the mPFC, respectively the units PLpv in the PL and ILpv in the IL, must be removed through the activation of somatostatin interneurons, respectively PLs and ILs (Courtin et al., 2014; McGarry and Carter, 2016; Lucas and Clem, 2018; Cummings and Clem, 2020).

The ITC neurons are activated by the IL via BA (BAp3) and have a feedback inhibitory projection to BA, driving the suppression of freezing (Berretta et al., 2005; Cho et al., 2013; Asede et al., 2015).

### 2.4. Hypotheses of the Model

We formulated some hypotheses to address the knowledge gaps in the experimental literature illustrated in section 1 and to complete the architecture of the model. These hypotheses, now illustrated in detail, could be tested in future empirical experiments.

The first hypothesis is that fear conditioning potentiates the connection between a subset of the LA neurons, that are responsive to the CS but not to the US, and the CeL-ON cells in CeL. While CeL is necessary for fear conditioning, the stimulation of the auditory thalamus does not induce a response in this structure (Li et al., 2013). This means that information concerning the CS is conveyed indirectly to CeL. Moreover, while most LA pyramidal neurons receive signals related to both the US and CS, some respond only to the CS (Romanski et al., 1993). We thus hypothesized that the CS signal reaches CeL through LA pyramidal neurons responsive to CS only, and that this pathway is potentiated during conditioning. Preliminary simulations suggested that LAp1-CeL-ON cannot be plastic, otherwise the simple presentation of US, and not the US-CS association, would drive the potentiation in LA-CeL pathway (data not shown).

The second hypothesis concerns the within- and between-session extinction. During extinction, there is a progressive recruitment of the BA neurons projecting to IL. This recruitment is caused by the release of endocannabinoids within BA and the DSI of the synapse connecting the interneurons BAcck to BAp2, the BA neurons projecting to the IL (Senn et al., 2014; Vogel et al., 2016). Interestingly, the IL is inactive during the first session of extinction, but it gets involved in it from the second session (Milad and Quirk, 2002). Given the transient nature of DSI/E (Kamprath et al., 2011), it is not clear how the IL neurons become responsive to the CS in the second session. Our hypothesis is that the temporary DSI occurring during the first extinction session enables a level of IL activity that is sufficient to drive synaptic plasticity at the synapses connecting BA to the IL (Vouimba and Maroun, 2011). Once the IL is activated, the firing of its pyramidal neurons recruit the BA neurons and the ITC, thus triggering the LTP at the BA-ITC connection known to occur due to extinction (Amano et al., 2010). During the second extinction session, the synapses are thus sufficiently strong to activate the IL and ITC even without the removal of the inhibition in BA induced by the endocannabinoids.

The third hypothesis concerns the LA-BA-CeM circuit. As previously shown (Corcoran and Quirk, 2007; Sierra-Mercado et al., 2011; Adhikari et al., 2015), the PL inactivation strongly reduces but does not completely abolishes freezing (see Figure 3 in Corcoran and Quirk, 2007 and Figure 2 in Sierra-Mercado et al., 2011). This can be due to an incomplete pharmacological blockade of the PL or to a parallel circuit connecting LA to CeM. Because it has been shown that input can reach CeM through different pathways departing from LA (Pitkänen et al., 1995; Savander et al., 1997), we chose the second possibility and hypothesized the existence of a parallel LA-BAp5-CeM circuit.

The fourth and last hypothesis concerns the mechanisms behind reinstatement. It was shown that fear reinstatement restores synaptic strength between the BA and the mPFC to a level observed before extinction (Vouimba and Maroun, 2011). We hypothesized that this effect is caused by an US-induced depression of the IL activity, in particular by the US exciting ILpv, that in turn inhibits ILp.

### 2.5. Synaptic Plasticity

Various works from the literature allowed us to establish which connections considered in the model are plastic and which are not (Figure 2). We used three different types of plasticity rules to reproduce LTP, LTD, and DSI/E (Table 1), and applied them at each step of the simulation.

**Table 1:** Plastic connections of the model.

**Connections**	**Plasticity type**	**References**
CS-pathway to LAp1	LTP	Rogan and LeDoux, 1995; McKernan and Shinnick-Gallagher, 1997; Rogan et al., 1997; Tsvetkov et al., 2002
LAp2 to CeL-ON	LTP	Li et al., 2013
PLp to BAp4	LTP	Vouimba and Maroun, 2011
PLp to BAp4	LTD	Vouimba and Maroun, 2011
BAp2 to IL	LTP	Vouimba and Maroun, 2011
BAp2 to IL	LTD	Vouimba and Maroun, 2011
BAp3 to ITC	LTP	Amano et al., 2010
BAcck to BAp2	DSI	Vogel et al., 2016
BAp4 to CeM	DSE	Kamprath et al., 2011
BAp5 to CeM	DSE	Kamprath et al., 2011

*The table shows the connections of the model undergoing plasticity, the type of such plasticity, and the supporting literature*.

LTP and LTD were implemented using the equation:

(4) $$\begin{array}{r} \Delta W=\eta \cdot \left(M-W\right)\cdot \left(Post-\phi \cdot \sigma \right)\cdot Pre \end{array}$$

where η is a constant (Supplementary Table 3), *M* is the maximum level that the connection weight can achieve (set to 3 times the initial level), *W* is the current connection weight, *Post* is the postsynaptic firing, σ is a threshold (Supplementary Table 3) and ϕ is the maximum firing of the neuron (Supplementary Table 1). The direction of the synaptic change depends on the post-synaptic element: if the postsynaptic firing is greater than the threshold the weight is increased, otherwise it is decreases (Gerstner and Kistler, 2002; Oliva et al., 2018).

DSI/E is triggered when postsynaptic neurons are depolarized for a sufficient amount of time to release endocannabinoids. In the model we set this time to 10 s based on empirical evidence (see Figure 2 in Kamprath et al., 2011). Another feature of DSI/E is their specificity, that in the brain is achieved in at least two ways. First, not every presynaptic terminal receiving endocannabinoids undergoes DSI/E. It has been shown that in BA cholecystokinin-positive basket cells contact both the IL projecting neurons and the PL projecting neurons, but only the IL connections can be depotentiated, maybe because of a different distribution in endocannabinoid receptors (Vogel et al., 2016). Thus, in the model only the synapse between BAcck and BAp2 is plastic. In CeM, where to our knowledge no such asymmetry has been described, all the excitatory synapses can be depotentiated (Kamprath et al., 2011). Second, some researchers observed that DSI/E occurs only to the active synapses where the presynaptic element is firing (Singla et al., 2007).

To implement DSI/E, we devised a plasticity rule that, after 10 s of postsynaptic depolarization, causes the change Δ*W* of the connection weight as follows:

(5) $$\begin{array}{r} \Delta W=-\eta \cdot W\cdot Pre \end{array}$$

where η is a multiplication constant (see Supplementary Table 3), *W* is the connection weight, and *Pre* is the presynaptic firing. To simulate the transient nature of DSI/E we returned the connection weights modified by DSI/E to their original values between the two sessions of extinction.

### 2.6. Parameters Setting, Search, and Sensitivity Analysis

As explained in section 2.2, most parameters of the model were set on the basis of the literature: the time coefficients of neural units, the tonic activity of the neurons, the constants and thresholds of plasticity, the firing thresholds, and the neuron maximum firing rates (Supplementary Table 1). However, the value of some parameters could not be found in the literature, so we set them following these criteria: (a) we used for BAcck the same electrophysiological properties of parvalbumin interneurons because it has been shown that cholecystokinin-positive basket cells and parvalbumin-positive basket cells have similar kinetics and capacity to inhibit pyramidal neurons (Veres et al., 2017); (b) to our knowledge, the identity of the interneurons that block the activity of parvalbumin interneurons when the US is delivered is not known (Krabbe et al., 2018): however, in LA the vasoactive intestinal polypeptide-expressing neurons (vip) are usually upstream the parvalbumin-positive basket cells (Rhomberg et al., 2018), so we treated LAvip as vip interneurons.

The search of the remaining parameters (the values of the synaptic weights and the learning rates) posed a hard challenge given the high number of target experiments to address. This challenge was caused by the fact that each parameter affected multiple experiments and that the search was done in parallel with the identification of the model architecture. To face this challenge, we used a manual parameter search methodology based on an interactive model simulator software (Supplementary Figure 1): (a) each parameter was associated to a *slider bar* of the program interface and the resulting activation of all units of the model were plotted in a dynamic graph: this allowed an immediate monitoring of the effect of parameter values on all the target experiments; (b) starting from a simple initial version of the model, the target experiments were introduced progressively and for each one the possible connection weight values, before and after learning, were searched with the sliders to fit all together the experiments considered that far.

The parameters found with this approach, reported in Supplementary Tables 2, 3 and used to reproduce all the target experiments, were also probed with a sensitivity analysis to check their robustness with respect to changes (Saltelli et al., 2008). An important difficulty of sensitivity analyses is the prohibitive computational cost when they are run with models having many “output variables,” in our case those related to the 25 target experiments. To face this problem, we followed a *One-factor-At-a-Time procedure* (Saltelli et al., 2008) where we investigated the effect on the target experiments of changing one parameter at a time while leaving the others to the values found with the previous procedure. More in detail, the procedure was as follows: (a) we changed each parameter at steps of 5% toward lower or higher values with respect to the “reference value” found with the procedure illustrated above, while all other parameters were left at their reference value; (b) we checked the first value of the parameter, found while moving away from the reference value, that caused the loss of at least one target experiment; (c) the criteria we used to consider an experiment as “lost” are indicated in detail for each experiment in Supplementary Table 4; in particular, to reproduce the experiments regarding the activation and deactivation of fear, extinction, and persistent neurons, we considered 20% of maximal activity as the threshold to consider a unit active; we considered CeM as expressing the fear behavior if it achieved at least 70% of its maximum activation; finally, in the experiments involving synaptic plasticity we considered LTP or LTD to have happened if they caused a modification of at least 20% of the post-synaptic potential (PSP). The results of that analysis allowed us to rank the parameters in terms of their importance for reproducing the experiments: the smaller the size of the possible range of variation of the parameter not causing the loss of any target experiment, the higher its importance for the results (i.e., the higher the sensitivity of the results to that parameter).

A relevant observation on the reproduction of the target data is due. Following other approaches using models with a level of abstraction as ours (Krasne et al., 2011; John et al., 2013; Moustafa et al., 2013; Carrere and Alexandre, 2015; Oliva et al., 2018), our aim was here to reproduce the target data qualitatively rather quantitatively. Nevertheless, we tried to be as much accurate as possible by defining the criteria illustrated above for the sensitivity analysis.

### 2.7. The Target Experiments

As mentioned in section 1, the model and our hypothesis were validated by reproducing 25 experimental findings (Supplementary Table 5). All the experiments reproduced in the simulations (Figures 3–**12**) used the same set of parameters, in particular those listed in the Supplementary Tables 2, 3.

**Figure 3:**
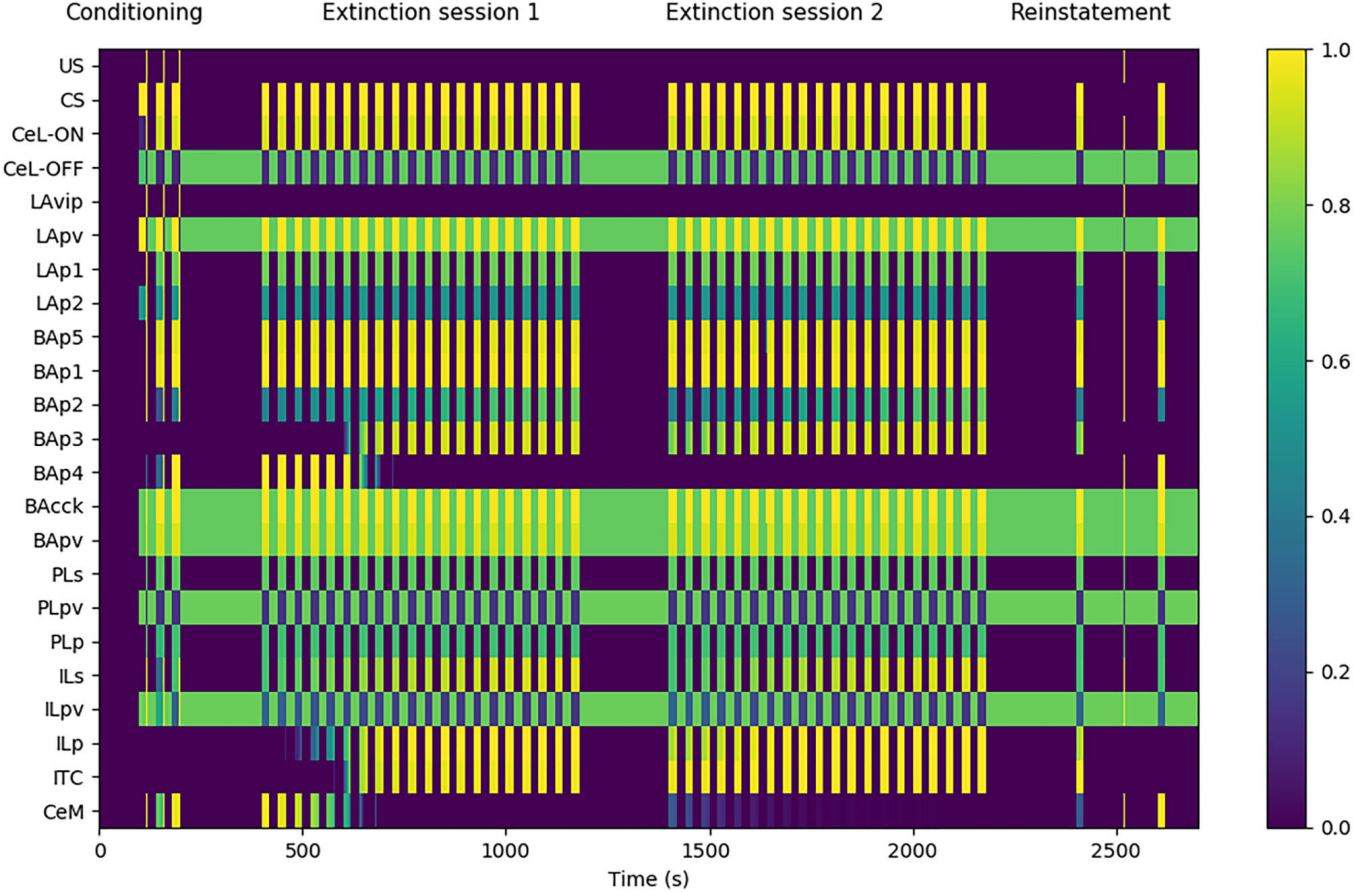
Model functioning during the tests. The graph shows in particular the activation of the model units, indicated in the y-axis, during a session of fear conditioning, formed by: 3 CS stimuli paired with a short US; two sessions of extinction, consisting in 20 CS stimuli each; and a session of fear reinstatement, involving a sequence of “CS, US, CS” stimuli. The x-axis represents the time of the sessions. The activation of CeM represents the expression of freezing.

The experimental protocol used in the simulations was formed by four sessions: a conditioning session, two extinction sessions, and a reinstatement session. Fear conditioning was performed by presenting three CS stimuli, each lasting 20 s, and by pairing them with an US for the last 0.5 s (Monfils et al., 2009). Fear extinction was performed by presenting the CS for 20 times (20 s each) without the US pairing (Monfils et al., 2009). A second 20-CS session was delivered to test the between-session extinction. Between the two extinction sessions, the weights updated by DSI/E incurred the spontaneous decay illustrated in section 2. Reinstatement was performed with the presentation of one 20 s CS, one unpaired 0.5 s US, and a last 20 s CS (Rescorla and Heth, 1975; Monfils et al., 2009).

In order to account for the experiments regarding potentiation/depotentiation of some pathways after fear conditioning, extinction, and reinstatement (McKernan and Shinnick-Gallagher, 1997; Amano et al., 2010; Vouimba and Maroun, 2011; Li et al., 2013), we measured synaptic changes in two ways. First, we compared the weight of the connection before and after the protocol. Second, to show how this weight modification would affect the unit response, we recorded the PSP induced by a presynaptic stimulation (30 ms of stimulation, stimulation adjusted to obtain a PSP below 10 mV).

To reproduce the experiment performed by Corcoran and Quirk (2007), consisting in the inactivation of the PL with tetrodotoxin (TTX) during fear conditioning, we set to zero all the connection weights of such area. In the test phase we restored the weights to their original value.

We simulated the optogenetic induction of LTP and LTD performed by Nabavi et al. (2014) by directly manipulating the connection weight from the CS-pathway to LAp1. In particular, to simulate the effects of LTP induction we tripled the weight of the connection, and to simulate the effects of LTP induction we replaced the weight with a zero value.

To reproduce the inactivation of LA and CeL during conditioning as done by Wilensky et al. (2006) and Ciocchi et al. (2010), we set the output of their units to zero.

To simulate the experiments described in **Figure 8C** and in Supplementary Figure 1, consisting in the substitution, during conditioning, of the US with the optogenetic depolarization of the LA (Johansen et al., 2010), we activated the units of LAp1 and LAp2 to their maximum value instead of delivering the US. To simulate the blocking of the extinction pathway, we set the output of BAp2 to zero in coincidence with LAp1 and LAp2 activation.

To simulate the IL lesion performed by Quirk et al. (2000) during extinction, we set the output of ILu, ILpv, and ILp to zero. To simulate the microstimulation of the IL performed by Vidal-Gonzalez et al. (2006) we set the output of ILp to its maximum value in coincidence to the CS.

Finally, to simulate the experiments performed by Marek et al. (2018) where the PL efferent connections toward the IL were activated or deactivated during fear extinction, we set the PLp output toward ILp to, respectively its maximum value or to zero during the CS delivery.

## 3. Results

### 3.1. Overall Behavior of the Model

Figure 3 shows that the model undergoes conditioning, extinction, and reinstatement as described in literature (Maren and Holmes, 2016). The three US-CS experiences of conditioning induce a progressive recruitment of the LAp1 unit projecting to BA. In turn, this activates the BA units BAp1 and BAp5 connected respectively to the PL and CeM and, to a lesser extent, the unit BAp2 connected to the IL. This lower activation is due to the fact that, while BAcck inhibits similarly BAp1 and BAp2 (as seen by Vogel et al., 2016; in our model both connections have a weight of 0.7, see Supplementary Table 2), the weight of the connection between LAp1 and BAp1 is higher compared to the one between LAp1 and BAp2 (7.0 vs. 5.0, see Supplementary Table 2). On the other hand, even though the weight of LAp1 to BAp5 is lower than the others (3.0, see Supplementary Table 2), the unit BAp5 does not receive inhibition.

During the first extinction session, the BAp2 unit of the BA increases its activity and so drives the activation of the ILp units of the IL to which it is connected, and these in turn activate BAp3 and the ITC. As a consequence, the activity of BAp4 progressively disappears under the inhibition of the ITC.

During conditioning and extinction the different elements of the model differentiate in the three classes of fear, extinction, and persistent units. Fear units, that exhibit an activity that is higher after conditioning and lower following extinction, are represented in BA by BAp4 units and in CeM by CeM units. Extinction units, that increase their activity during the repeated presentation of unpaired CS stimuli, are represented by BAp3 and BApv2 units in BA, the ITC unit, and the ILp unit in IL. Finally, persistent units, that have a high activity after both conditioning and extinction, are represented by the LAp1 unit in LA, the BAp1, BAp5, BAcck, and BApv units in BA, the CeL-ON unit in CeL, and the PLp unit in PL.

During the second extinction session, there is a residual activity of CeM that fades away during the session but re-appears in the following first CS stimulus of the reinstatement due to the forgetting of the connection weights involved in the endocannabinoids-induced DSI/E. This result is in line with the results reported by Kamprath et al. (2011), showing a persistent residual freezing after the extinction sessions. In the model, the residual activity is driven by the persistent activation of the BAp5 unit of BA that directly projects to CeM.

The reinstatement test shows that the inhibition exerted by the extinction circuit is easily reversible and the CS-conditioned CeM activity can be reinstated with a single US presentation. In particular, reinstatement induces the reappearance of firing in the fear unit BAp4, together with the shutdown of the extinction units BAp3, ILp, and ITC.

### 3.2. Synaptic Plasticity During Conditioning, Extinction, and Reinstatement

**Figure 4:**
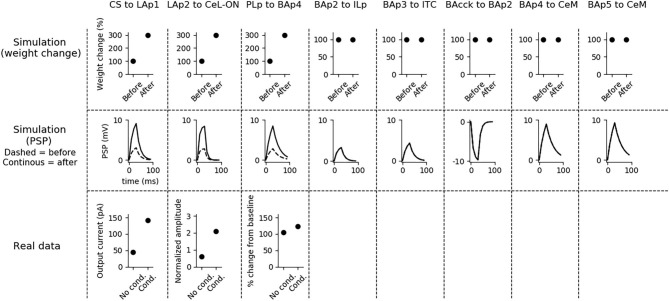
Changes of the connection weights and PSP before and after fear conditioning. First row: simulated connection weights before and after fear conditioning. Second row: simulated effect of a 30 ms presynaptic stimulation on the PSP of the plastic connections of the model (the presynaptic stimulation was adjusted to obtain a change of maximum 10 mV in the postsynaptic unit). Third row: available real data from literature, showing an LTP respectively in the output current evoked in LA neurons by a stimulation of the CS-pathway (McKernan and Shinnick-Gallagher, 1997), in the normalized current evoked in CeL-ON neurons with a stimulation of LA (Li et al., 2013), and in the response of BA to the mPFC input (Vouimba and Maroun, 2011). The other simulated synapses shown here to be stable have never been reported in the literature to undergo LTP or LTD during fear conditioning.

Aiming to understand how synaptic strength changes during conditioning, we measured synaptic weights before and after conditioning. Moreover, to show how the postsynaptic responses changes following conditioning, we recorded the PSP of the units undergoing plasticity using the procedure illustrated in section 2. The results are shown in Figure 4.

Fear conditioning enhances the PSP evoked by the CS-pathway in LAp1, by LAp2 in CeL-ON, and by PLp in BAp4. These changes reflect the potentiation observed in acute slices and *in vivo* after a protocol of fear conditioning (McKernan and Shinnick-Gallagher, 1997; Tsvetkov et al., 2002; Schafe et al., 2005; Vouimba and Maroun, 2011; Li et al., 2013).

**Figure 5:**
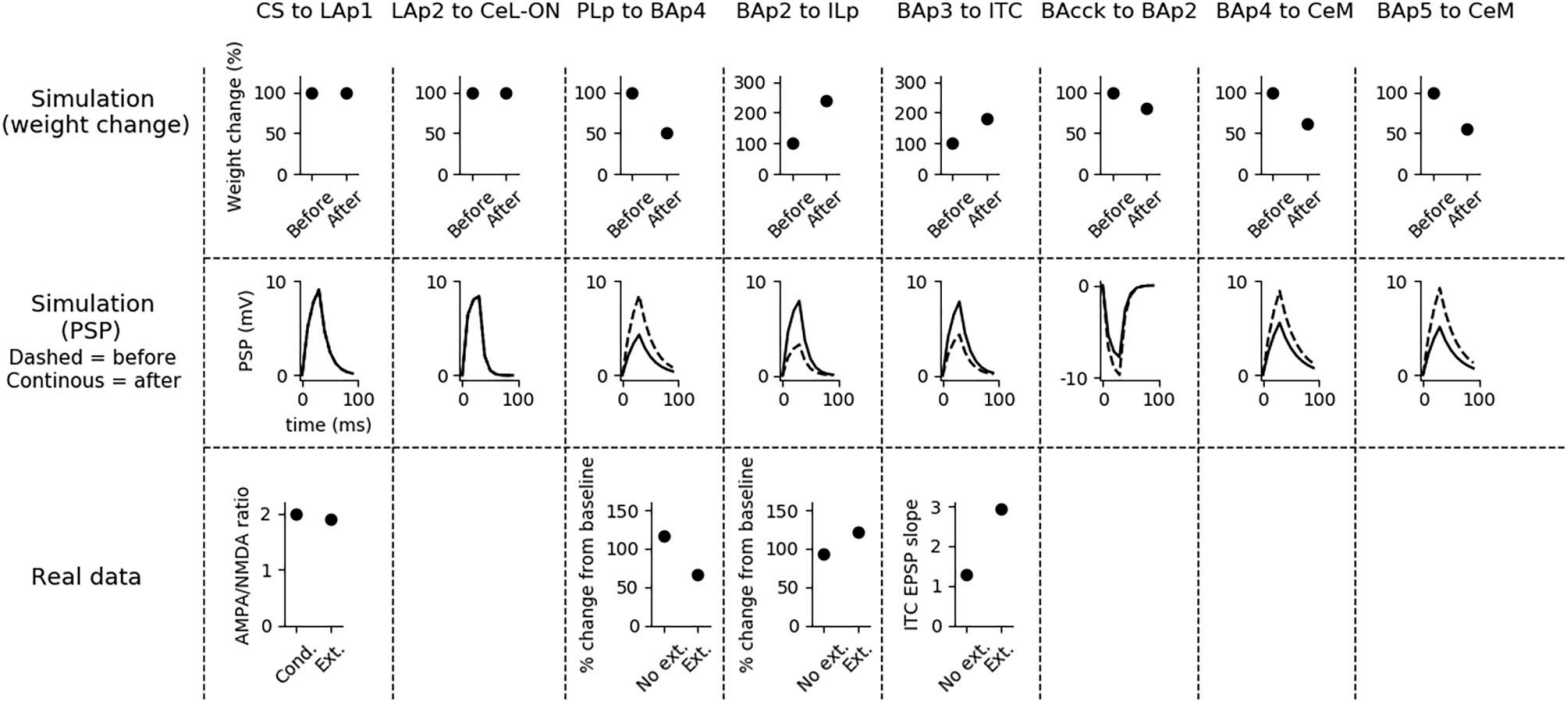
Changes in connection weights and PSP before and after fear extinction. First row: simulated connection weights before and after fear extinction. Second row: simulate effect of a 30 ms presynaptic stimulation on the PSP of the plastic connections of the model (the presynaptic stimulation was adjusted to obtain a change of maximum 10 mV in the postsynaptic unit). Third row: real data (Vouimba and Maroun, 2011) showing that, as in simulation, the mPFC-induced activation of BA decreases after extinction whereas the BA-induced activation of mPFC increases; moreover, as reported by Amano et al. (2010), BA stimulation evokes a higher EPSC slope in ITC neurons after extinction. On the other hand, AMPA/NMDA ratio at the synapse between the CS pathway and LA does not change (Kim and Cho, 2017). The other simulated synapses shown here to be stable have never been reported in the literature to undergo LTP or LTD during fear extinction.

Fear extinction (Figure 5) does not change the weights of the CS-LAp1 (Clem and Huganir, 2010; Kim and Cho, 2017), and LAp2-CeL-ON connections, but reverts the potentiation induced on the connection PLp to BAp4 by fear conditioning. Moreover, extinction potentiates the connections from BAp2 to ILp, and from BAp3 to ITC. This is in line with what reported in Clem and Huganir (2010), Kim and Cho (2017), Vouimba and Maroun (2011), Cho et al. (2013), and Amano et al. (2010).

The three connections sensitive to DSI/E, namely BAcck-BAp2, BAp4-CeM, and BAp5-CeM, are transiently weakened at the end of the extinction session. This implies that BAp2 is more active, once relieved from the inhibition of BAcck (as shown in Figure 3), and that DSE reduces the capability of fear and persistent units to activate CeM. Note that no real data corresponds to these synaptic modification because DSI/E has never been examined *in vivo* after fear extinction, given the transient nature of endocannabinoid action, so this is a prediction of the model.

**Figure 6:**
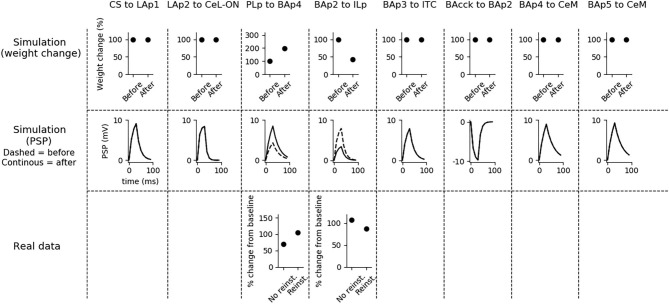
Changes in connection weights and PSP before and after fear reinstatement. First row: simulated connection weights before and after fear reinstatement. Second row: simulated effect of a 30 ms presynaptic stimulation on the PSP of the plastic connections of the model (the presynaptic stimulation was adjusted to obtain a change of maximum 10 mV in the postsynaptic unit). Third row: real data from Vouimba and Maroun (2011) showing that, as in simulation, inputs from the mPFC to BA increase after reinstatement, while those from BA to the mPFC decrease, restoring the synaptic strength to conditioning levels. The other synapses shown here to be stable have never been reported in the literature to undergo LTP or LTD during fear reinstatement.

Fear reinstatement changes the strength of the synapses between the mPFC and BA units (Figure 6). In particular, PLp-BAp4 and BAp2-ILp connections return to pre-extinction conditions, as shown in Vouimba and Maroun (2011), thus allowing PLp to activate BAp4 and preventing the recruitment of the extinction pathway by BAp2.

An overview of the neural units of the model that are activated/inactivated by a CS presentation after fear conditioning, extinction, and reinstatement, as well as a graphical representation of the potentiation/depotentiation of the connections, is also given in Supplementary Figures 2–4.

### 3.3. Conditioning: Specific Experiments

**Figure 7:**
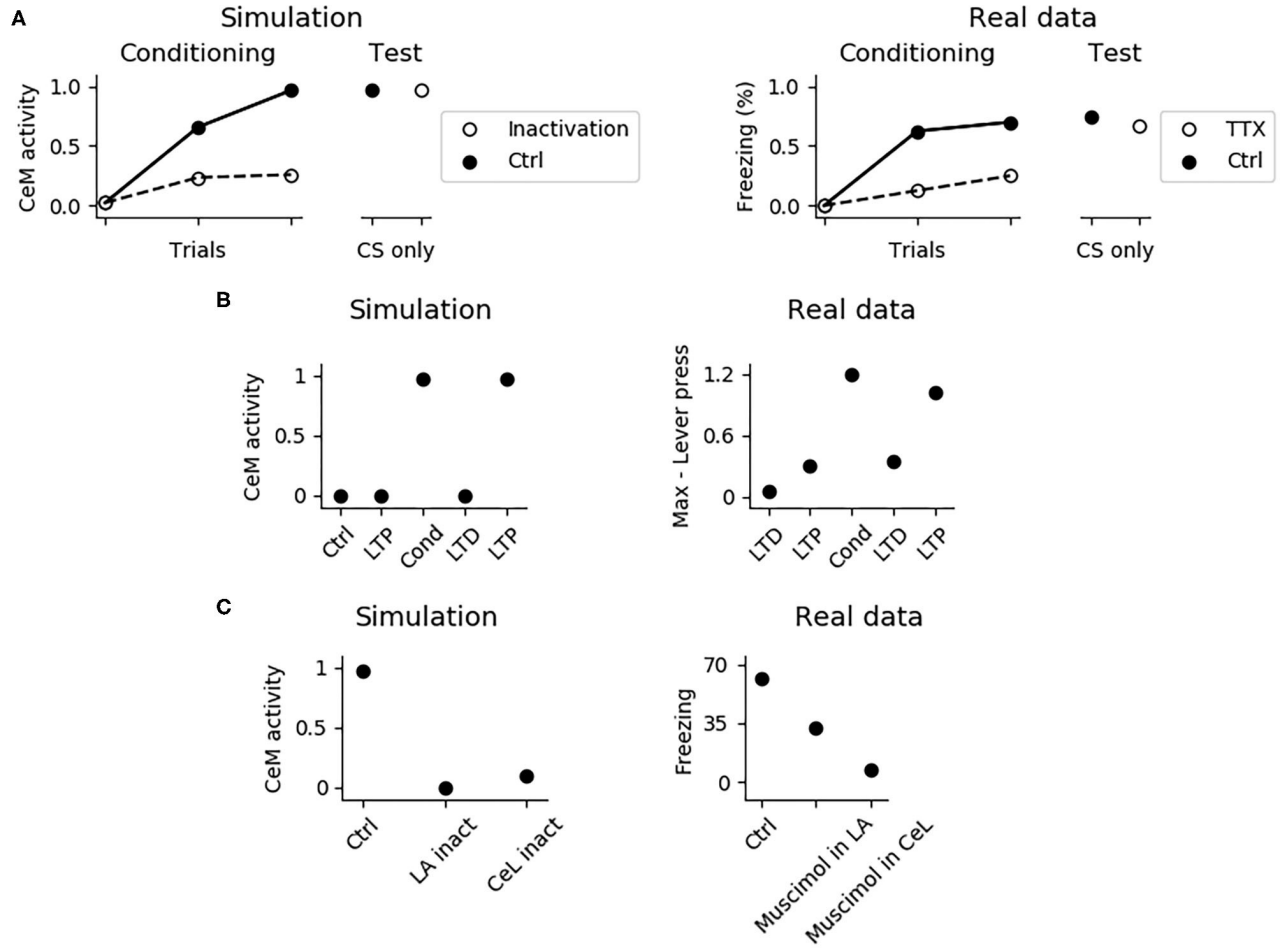
Effects of various manipulations of the model during conditioning. **(A)** When the PL is inactivated, mimicking the action of TTX, CeM activation is impaired during the CS delivery in the three trials of fear conditioning; this manipulation does not affect fear conditioning, as shown in a successive test phase, where the PL is restored. The simulation correctly reproduces real data reconstructed from Corcoran and Quirk (2007). **(B)** CeM activity after the CS delivery in the control model (Ctrl), in the model where the CS-pathway to LA was potentiated (LTP), conditioned (Cond), depotentiated (LTD), and repotentiated (LTP). The simulation mimics the findings of Nabavi et al. (2014). **(C)** If LA (La inact) or CeL (CeL inact) are inactivated during conditioning, in the successive test phase the CS fails to activate CeM. Real data reconstructed from Wilensky et al. (2006) showing that the GABAa agonist muscimol, injected to inactivate LA or CeL during training, strongly impairs fear conditioning in the test phase.

It has been shown that the PL is important for fear expression but not for fear conditioning. In particular, its inhibition with TTX reduces freezing to 40% during conditioning, but this effect disappears during the test phase, when the functionality of the PL is re-established (Corcoran and Quirk, 2007). The system correctly model these findings (Figure 7A), showing that the activity of CeM is reduced during conditioning, when the PL is inactivated, but it is indistinguishable from control conditions after the PL restoration. Thus, CS-US association is established correctly even without a functioning PL. This means that the most important regions for conditioning are located in the upstream circuit of the PL, consisting of LA and CeL.

The model accounts for the experiments reported by Nabavi et al. (2014) showing that synaptic plasticity of the connection between the CS-pathway and LA is necessary but not sufficient to establish fear conditioning. In particular, Figure 7B shows that increasing at its maximum level the weight of the connection between the CS-pathway and LAp does not cause a CS-induced CeM activation. However, after a conditioning session CeM fires in response to the CS: after this, the CeM response can be turned off through depotentiation of the pathway and can be turned on again with re-potentiation. This happens because, beside LA, also CeL is necessary for conditioning. In this regards, Figure 7C shows that when CeL is inactivated, conditioning fails. In agreement with this, Ciocchi et al. (2010) showed that CeL contains neurons receiving inputs from LA that, after conditioning, respond to the CS with an increase or with a decrease in firing. In the model, those neurons are modeled by the units CeL-ON and CeL-OFF.

**Figure 8:**
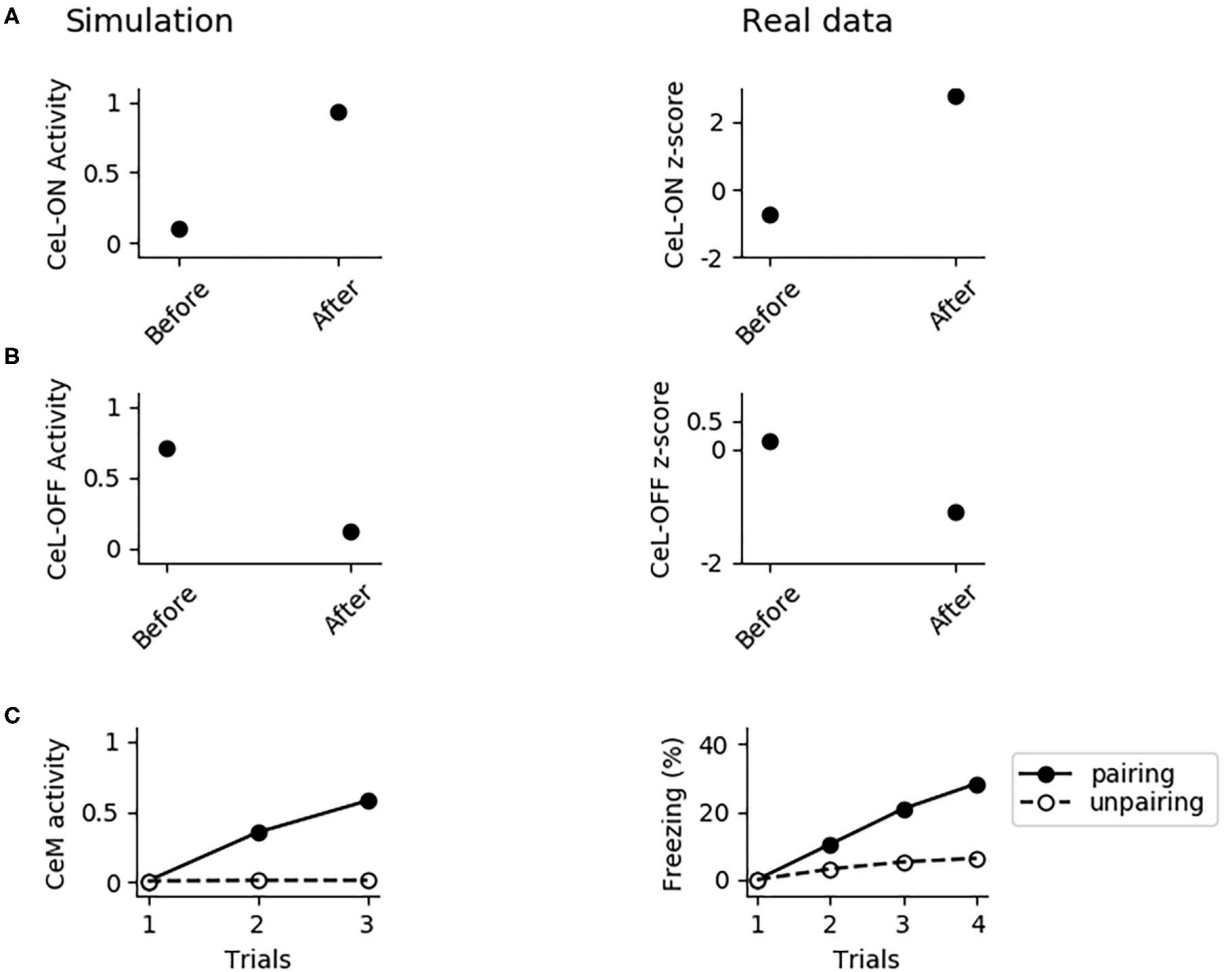
The involvement of CeL in conditioning. **(A)** Activity of CeL-ON and **(B)** CeL-OFF when the CS is delivered before and after conditioning, and comparison with data from Ciocchi et al. (2010). **(C)** CeM activity during the CS delivery in the three trials of conditioning, when the CS is paired with a maximum depolarization of PLp1 and PLp2; we used a similar protocol as control where we unpaired the CS and depolarization.

In Figure 8, we further investigated the role of CeL in conditioning. We observed that conditioning causes CeL-ON unit to become responsive to the CS and induces the shutdown of the CeL-OFF unit (Figures 8A,B). Thus overall the model reproduces the evidence for which LA and CeL are both necessary but none of them is alone sufficient for conditioning. Indeed, LA induces the activation of the fear and persistent units in BA, that drive CeM depolarization, but CeL exerts a brake that must be removed in order to obtain CeM firing.

As discussed above, given that the US and CS signals reach CeL through LA, it should be possible to induce conditioning by pairing the CS with a depolarization of LA, as reported by Johansen et al. (2010), using an optogenetic induced activation of LA instead of an US. Indeed, the CS-pathway should potentiate if the postsynaptic neurons in LA are depolarized at the same time. Moreover, if LA conveys both the US and CS signals to CeL, the depolarization of LA projection units should reproduce the same effects of fear conditioning. Indeed, in the model CeM activity increases as we pair the CS with a depolarization of LAp1 and LAp2 that substitutes the US. As shown in Figure 8C, the control test, consisting in the CS delivery without pairing with the LA depolarization, does not show any conditioning. Interestingly, with the pairing protocol of CS and depolarization we could only obtain a weaker conditioning, compared to what observed using the classic protocol (cf. Figure 7A with Figure 8C).

**Figure 9:**
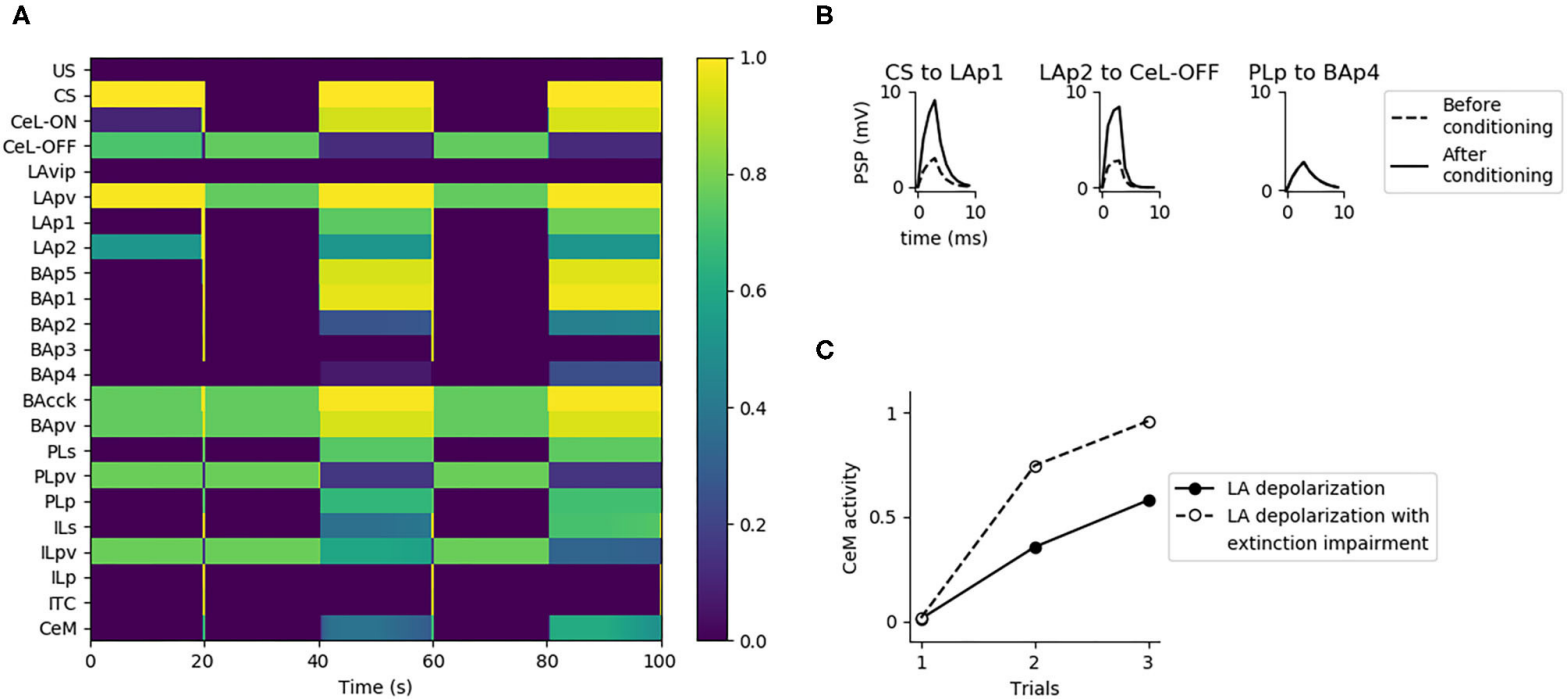
Substitution of US with LA activation during conditioning. **(A)** When LAp1 and LAp2 are activated at they maximum, substituting the US during the three trials of conditioning, the extinction circuit composed by BAp2, ILp, BAp3, and ITC is engaged. This causes the suppression of the activity of BAp4. **(B)** When LA activation is used instead of the US there is potentiation of only two of the three connections that should undergo LTP during conditioning. **(C)** Fear conditioning obtained with LA activation, in control condition (Ctrl, black dots) or when the extinction pathway is inactivated (BAp2 inactivation, white dots).

To better understand why conditioning is weaker if the US is substituted with LA depolarization, as also observed in Johansen et al. (2010), we analyzed the activation of the units composing the model while delivering the three trials of conditioning (Figure 9A; see also Supplementary Figure 5, that shows the active/inactive units during conditioning). We observed that when the US is substituted with a massive depolarization of the LA, the unit BAp2 projecting to IL is maximally active. This, in turn, drives the activation of the extinction units BAp3 and ITC, that strongly reduces the firing of the fear unit BAp4. The consequence is the incomplete potentiation of the fear pathway: the connections from the CS-pathway to LAp1 and from LAp2 to CeL-ON undergo LTP, but the connection from PLp to BAp4 remains the same, inducing a lower conditioning (Figure 9B). A full conditioning can be obtained if the extinction pathway is inactivated while LAp1 and LAp2 are depolarized in substitution to the US (Figure 9C).

### 3.4. Extinction: Specific Experiments

**Figure 10:**
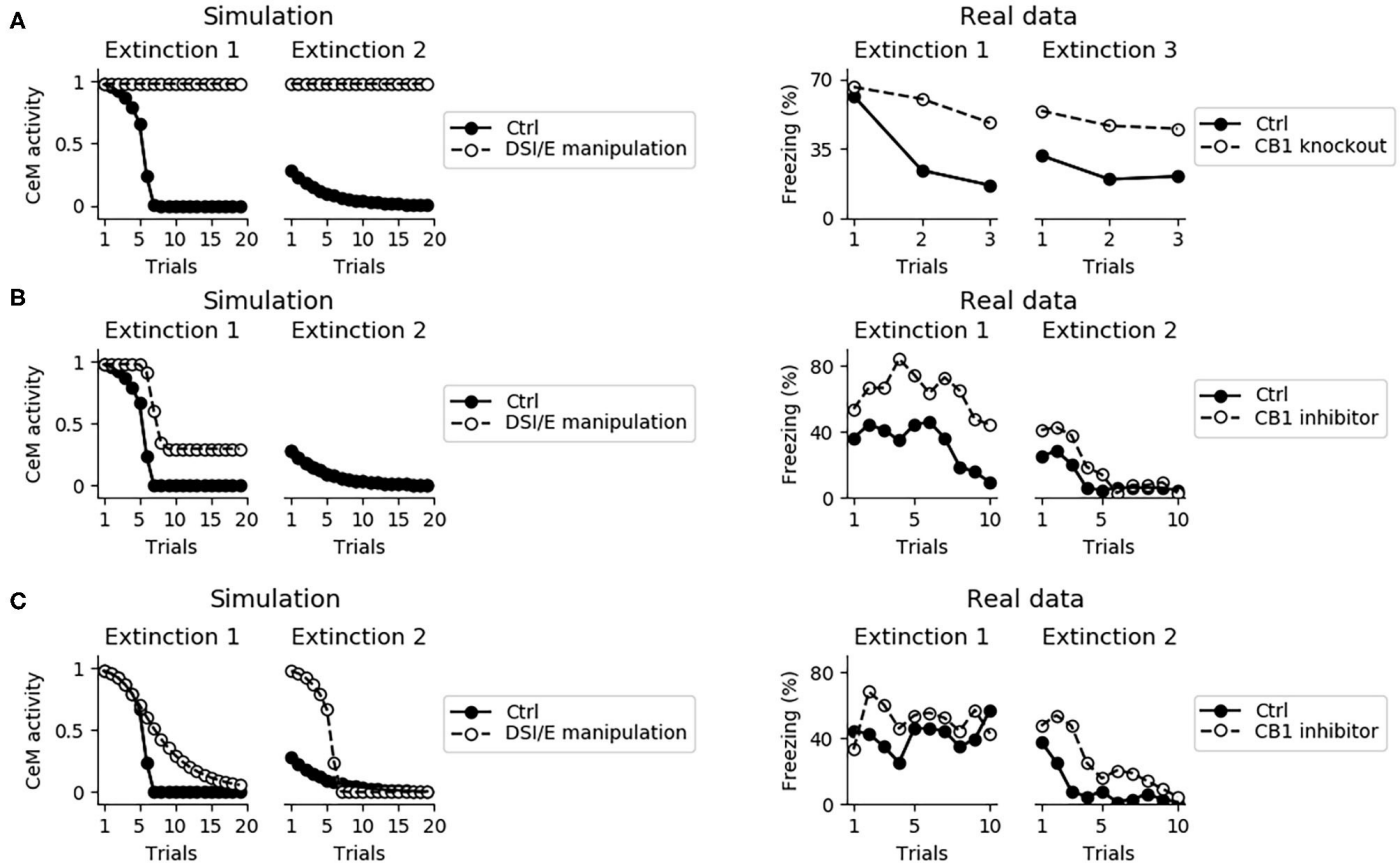
Effects on extinction of the manipulation of the DSI/E. **(A)** CeM activity during the two sessions of extinction in the control model (black dots) and in a model where DSI/E is inactivated (white dots) in the whole amygdala. As shown in real data from Marsicano et al. (2002) in the right graph, knockout mice for the endocannabinoid receptor CB1 are severely impaired in both within- and between-session extinction. **(B)** CeM activity during the two sessions of extinction in the control model and in a model where DSI/E is inactivated in CeM during the first session. In agreement with data from Kamprath et al. (2011), extinction is impaired in the first session but is spared in the second session. **(C)** CeM activity during the two sessions of extinction in the control model and in a model where DSI/E is inactivated in BA during the first session. The control and DSI/E-inactivated models have comparable levels of extinction in the first session, but the manipulated models shows a deficit in between-session extinction, in agreement with real data (Kamprath et al., 2011).

Endocannabinoids play a central role in between- and within-session extinction (Marsicano et al., 2002; Kamprath et al., 2006, 2011). The model captures this because when DSI/E is impaired in the whole amygdala the fear behavior does not disappear during two consecutive extinction sessions (Figure 10A).

In the model, DSI/E is present in CeM and BA with different roles: in BA it reduces the inhibition on the unit that projects to IL, whereas in CeM it depresses the excitatory inputs to the output unit CeM. Blockade of DSI/E in CeM or in BA during the first session of extinction gives results coherent with the experiments reported by Kamprath et al. (2011). In particular, when DSI/E is abolished in CeM the model responds with a poor extinction in the first extinction session but gives results indistinguishable from the control in the second extinction session (Figure 10B). On the other hand, if the blockade of DSI/E is performed in BA, the model extinguishes correctly but is impaired in between-session extinction (Figure 10C).

**Figure 11:**
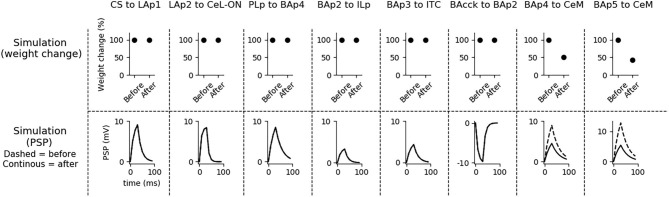
Effects on synaptic plasticity of DSI blockade in BA during extinction. If DSI is inactivated during extinction, plasticity is severely compromised at the synapses PLp-BAp4, BAp2-ILp, BAp3-ITC, and BAcck-BAp2 (compare these results with Figure 4).

The activation of the IL and in particular of ILp neurons, induced by DSI in BA during the first extinction session (Figure 3), is necessary for the formation of the extinction circuit. Indeed, blocking DSI/E in BA impairs the potentiation of connections on the postsynaptic extinction units ILp, BAp3, and the ITC. Moreover, it prevents the depotentiation of the input driven by PLp on the fear unit BAp4 (Figure 11).

**Figure 12:**
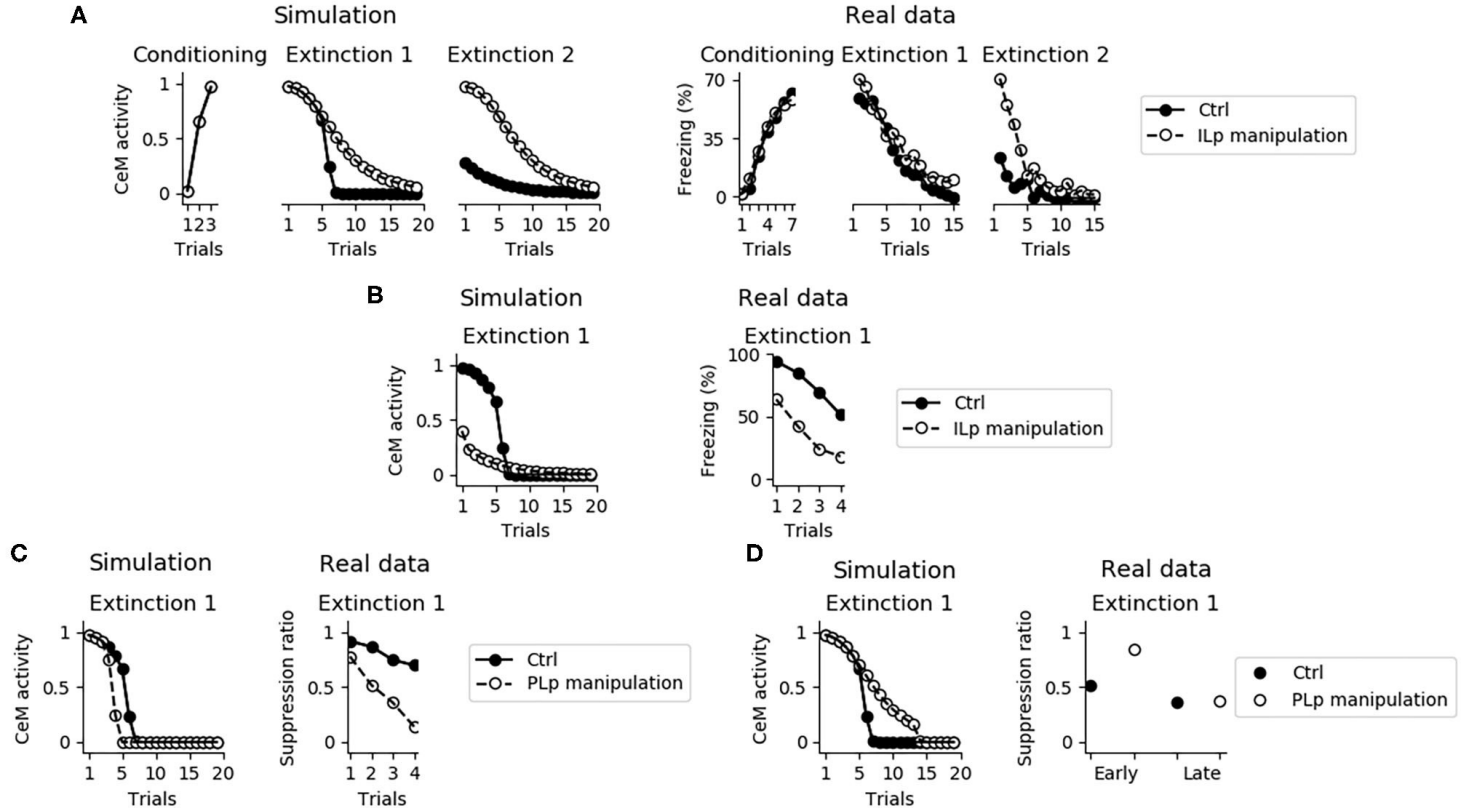
Effects of the IL and PL manipulation on extinction. **(A)** The inactivation of ILp does not influence conditioning and the model shows extinction during the first session, as observed by Quirk et al. (2000). On the other hand, the model shows that the absence of a functioning IL causes a deficit in between-session extinction, as shown in Quirk et al. (2000). **(B)** Conversely, when the IL is stimulated extinction is faster, in accordance with data presented by Vidal-Gonzalez et al. (2006). **(C)** The connection between the PL and IL controls the speed of fear extinction, as demonstrated in real data from Marek et al. (2018). In particular, if PLp-ILp connections are stimulated then fear extinction occurs earlier. **(D)** Instead, if PLp-ILp connections are inactivated then extinction takes longer.

Despite its fundamental role in the retention of extinction from the first extinction session to the second one, the IL is not necessary for the expression of fear conditioning and for the within-session extinction (Figure 12A; Quirk et al. 2000, Do-Monte et al. 2015, Kim et al. 2016, Bloodgood et al. 2018). The reason is that the within-session extinction depends on DSE of the connections between BA and CeM. On the other hand, the between-session extinction requires the potentiation of the BAp2 to ILp and BAp3 to ITC connections, that is impaired when IL is inactivated (see also Supplementary Figure 6).

Figure 12B shows that if ILp is activated at its maximum level simultaneously with the CS presentation, extinction during the first session occurs much faster, reproducing what observed by Vidal-Gonzalez et al. (2006) and Adhikari et al. (2015).

It has been recently pointed out that the PL is a major source of input for the IL, such that stimulation or inhibition of the PL terminals projecting to the IL affect the number of trials needed to obtain extinction (Marek et al., 2018). We reproduced these results with the model. In particular, Figure 12C shows that a maximum activation of the connection from PLp to ILp speeds up extinction whereas Figure 12D shows that its inhibition impairs early but not late extinction during the first extinction section.

### 3.5. Sensitivity Analysis

Some of the parameters of the model, in particular the weights of the connections and the synaptic plasticity coefficients, were obtained through the incremental systematic search illustrated in section 2. The robustness of the model with respect to the parameters found in such a way was probed through the sensitivity analysis illustrated in the same section.

Supplementary Figure 7 shows the results on the sensitivity to changes of the synaptic weights. The graph shows that some parameters (15 out of 41, i.e., 37%) should have a value within an interval smaller than ±5% in order to replicate all the target data. Other 16 parameters (39%) can vary in a wider range, up to +80% for the US-LAp1 connection and −35% for the BAp4-CeM connection. Our initial model included a neuron BApv2, getting a synaptic connection from ILp and projecting to BAp3 (Cho et al., 2013), and also a US input directly reaching CeM (Paré et al., 2004). However, the sensitivity analysis showed that the weights of these three connections could be set to zero without affecting the model capacity to reproduce the target experiments. We thus removed these connections and the neuron BApv2 from the model. These connections, while not necessary for the experiments targeted here, might be relevant to reproduce other experimental data. Finally, 9 parameters (22%) can span a very large range without impairing the results. In particular, ILp-BAp3, ILp-ITC, LAvip-PApv, and US-LAvip connections can be increased, before the model fails to fit at least one experiment, to, respectively +790, +225, +620, and +485%; instead, ITC-BAp4, BAp3-ITC, US-ILu, US-CeM, and ILp-BApv2 connection weights have been increased up to +1,000% without loosing a good fit of any target experiment, thus showing that the model is very robust to an increase of such parameters (of course, biologically the strength of such connections has an upper bound).

Supplementary Figure 8 shows the results of the sensitivity analysis of the plasticity parameters. Overall, the values of these parameters can vary more than the connection weight parameters. None of the parameters can be set to −100% (amounting to no plasticity), showing that all the plasticity mechanisms of the model are necessary to reproduce all the target experiments. Most of the parameters can be increased to a very high value. In particular, the model fails to fit at least one experiment only if DSE BAp5-CeM, DSE BAp4-CeM, and DSI BAcck-BAp2 reach, respectively +215, +425, and +390%, while it is still fully successful when LTP CS-LAp1, LTP BAp2-ILp, LTP BAp3-ITC, LTP LAp2-CeL-ON, and LTP PLp-BAp4 are increased up to +1,000%.

## 4. Discussion

### 4.1. Hypotheses of the Model

We formulated four specific hypotheses to build the proposed model able to integrate the information from the literature on freezing. The first hypothesis addresses the fact that currently it is not known how the US and CS reach the CeL, a nucleus important for fear conditioning. CeL contains CeL-OFF neurons that inhibit, as a brake, the CeM neurons projecting to the periaqueductal gray matter (Haubensak et al., 2010; Li et al., 2013). For conditioning to take place this brake must be removed by the activation from LA toward CeL-ON neurons. At the same time, thalamic and cortical afferents carrying the CS information should trigger CeM response passing through a relay in BA. Given that the US can be substituted by LA stimulation (Johansen et al., 2010), the US too must be transmitted to CeL through LA. It has been shown that in LA there is a subgroup of neurons that respond to the CS but not to the US (Romanski et al., 1993). We thus hypothesized that the CS is transmitted through LA to CeL-ON neurons through such US-independent subgroup of LA neurons. This connection from CS-only LA neurons to CeL-ON neurons should correspond to the connection that was found to be potentiated by Li et al. (2013). As shown in the results presented here, this hypothesis allowed the model to induce an LTP when both US and CS are present (Figure 4) and to reproduce the conditioning-induced CeL-ON activation and CeL-OFF inactivation (Figures 8A,B) shown by Li et al. (2013).

The second hypothesis is that DSI has a causal role in recruiting the IL during fear extinction. It is known that during extinction BA neurons projecting to IL are progressively engaged (Senn et al., 2014). We thus hypothesized that endocannabinoids play a key role in allowing this process. Indeed, blocking endocannabinoids in BA gives a deficit in extinction recall: this is a result very similar to the one obtained with the IL inactivation or with the impairment of the LTP consolidation within it (Quirk et al., 2000; Santini et al., 2008; Kamprath et al., 2011; Sepulveda-Orengo et al., 2013; Criado-Marrero et al., 2014; Bloodgood et al., 2018). This suggests that the IL and BA endocannabinoids are somehow part of the same pathway. Interestingly, Vogel et al. (2016) discovered that interneurons in BA inhibit both PL and IL projecting neurons, but only connections to IL projecting neurons undergo endocannabinoid-mediated DSI. Therefore, we hypothesized that DSI in BA, which is a transient process, functions like a trigger for a downstream persistent process (the activation of the extinction circuit through LTP) orchestrated by the IL.

As far as we know, only two computational models have analyzed the involvement of the endocannabinoid system in the extinction of fear. These models are based on very different hypotheses from ours. The model of Anastasio (2013) performed a computational search of the synapses whose depotentiation is compatible with a decrease in the activity of CeM. The search was limited to GABAergic synapses but it has been shown that also glutamatergic synapses in amygdala are affected by endocannabinoid-induced depotentiation (Kamprath et al., 2011). Moreover, endocannabinoids exert different effects, depending on the subregion of the amygdala considered (Kamprath et al., 2011).

A second computational model proposes that the cholecystokinin-positive interneurons expressing endocannabinoid receptors act by repressing the extinction neurons in BA (Bennett et al., 2019). During extinction, the input from LA and IL on extinction neurons allows a sufficient depolarization to activate the endocannabinoid system. This, in turn, triggers DSI on the connection between cholecystokinin-positive interneurons and extinction neurons, potentiating the CS-induced activation of the extinction neurons. In this model, endocannabinoids affect the within- but not the between-session extinction in line with data from Plendl and Wotjak (2010). However, in other experimental works it has been shown that the endocannabinoid system manipulation compromises not only the within-session but also the between-session extinction (Marsicano et al., 2002; Kamprath et al., 2011). In particular, it was observed (Kamprath et al., 2011) that a transient BA endocannabinoid blockade on the first session does not influence the within-session process, but significantly reduces extinction during the following sessions. In the model proposed here, coherently with the view of Marsicano et al. (2002) and Kamprath et al. (2011), both within- and between-session extinction are compromised by endocannabinoids (Figure 10). This is possible because in the model the within-session extinction relies mainly on the action of DSI/E at the level of CeM, while the between-session extinction depends on the DSI/E in BA that slowly recruits the IL, allowing the build-up of LTP in the synapses between BA and the IL and, downstream, between BA and the ITC. A possible way to reconcile our model with the model of Bennett et al. (2019) and the results from Plendl and Wotjak (2010) is suggested in the latter work itself. In particular, they observed that both the within- and between-session extinction are influenced by the protocol used for the CS administration. In particular, with a protocol consisting of the presentation of multiple 20-s CS stimuli separated by a variable time interval, instead of using permanent tones, it is possible to obtain a successful within- and between-session extinction even with mice knockout for the endocannabinoid receptor (Plendl and Wotjak, 2010). Interestingly, in support of this possibility the cFos activation in the IL during extinction is significantly affected by the protocol of CS presentation, by endocannabinoid inhibitors, and by the interaction of both factors (Plendl and Wotjak, 2010). We thus propose that, even in the absence of a functional endocannabinoid system in BA, the IL could be activated by other experimental protocols not considered here. Future work might aim to modify the model regarding its sensitivity to the CS administration timing to account for its differential effects on extinction dynamics.

Our third hypothesis was that the excitatory input from LA reaches CeM through a direct connection from BA, alongside a relay in the PL and another relay in CeL. The necessity of this alternative route from LA to CeM emerged because the blockade of the PL with TTX reduces, but does not completely erases, fear expression (Corcoran and Quirk, 2007; Sierra-Mercado et al., 2011). In our model, the unit of BA that brings the excitatory input from LA directly to CeM is represented by BAp5. We built BAp5 as a unit subjected to DSE, thus contributing to the within-session extinction (Figure 5). On the other hand, BAp5 is not a target of the projection from the extinction circuit and is thus not influenced by the within-session extinction. This circuit architecture allows the reproduction of the data from Kamprath et al. (2011) where it was observed that after the first extinction session a residual freezing remains at the beginning of the following sessions two and three and that this freezing fades within- but not between-sessions (see Figure 5 in Kamprath et al., 2011).

The fourth hypothesis we proposed is that aversive unconditional stimulus inhibits the IL, thus driving a mismatch between the activity of the units BAp2 and ILp and a consequent LTD of the connections linking them. Some experimental research support this hypothesis. It has been shown that pyramidal neurons in the IL, but not in the PL, are very sensitive to acute stressors and respond with dendritic retraction (Izquierdo et al., 2006; Moench et al., 2016). Moreover, Hitora-Imamura et al. (2015) showed that the US activates dopaminergic projection from ventral tegmental area to the IL, which in turn activates dopamine D1 receptors in the IL resulting in a lower activity. Under this hypothesis, the model showed that a single, unpaired US induces the depotentiation of the BAp2-ILp2 connection, in agreement with what found by Vouimba and Maroun (2011). In this condition, the model also produces the potentiation of the connection between PLp and BAp4 (Figure 3), a result confirmed by Vouimba and Maroun (2011).

The four hypotheses, leading to the correct reproduction of the 25 experiments addressed here, could be validated in future empirical experiments by testing various predictions they suggest. We present here three of these predictions: (1) fear conditioning should lead to LTP at the connection between CS-responsive LA pyramidal neurons and CeL-ON neurons; (2) pharmacological blockade of CB1 receptors in BA impairs the recruitment of the IL, so that during the second session of extinction the IL pyramidal neurons should not fire after a CS presentation; (3) electric shock should result in the silencing of the IL pyramidal neurons projecting to BA.

### 4.2. Comparison With Other Computational Models

In the last years, several computational models of different types have been proposed to study fear conditioning and extinction: from bottom-up realistic models, that emulate the biophysical properties of neurons in detail (e.g., Kim et al., 2013), to more abstract top-down models, aimed at providing functional explanations of behavioral data (e.g., Krasne et al., 2011). Given this large production, we focus here on the models, listed in Supplementary Table 6, that more closely address the phenomena studied here and that have been most discussed in the literature (Nair et al., 2016; Li, 2017).

Methodologically, in terms of modeling abstraction level the model presented here falls between the two approaches used by Krasne et al. (2011) and Kim et al. (2013). In particular, similarly to Krasne et al. (2011), it abstracts on the representation of neurons, reproduced in groups through leaky units, and focuses on explaining several target experimental behaviors; on the other side, similarly to Kim et al. (2013), it uses a very accurate biologically-constrained network circuitry that might underpin these behaviors.

Among the more realistic bottom-up models we can mention those proposed by Li et al. (2009) and Kim et al. (2013) as examples. These models simulate single neurons in detail, with equations reproducing different ionic currents and some of the underlying electro-chemical mechanisms. This allows the models to correctly reproduce the firing patterns of three subgroups of LA pyramidal neurons having a different firing rate adaptation in response to the CS. The advantage of using “bottom-up” models is a higher focus and the possibility of reproducing the target experiments in much detail. However, their computational complexity impinge on the number or regions that can be simulated together.

On the other side, “top-down” models that abstract over biological details have instead the advantage of the capacity to reproduce behavioral data (Burgos and Murillo-Rodríguez, 2007; Krasne et al., 2011; Anastasio, 2013; John et al., 2013; Moustafa et al., 2013; Carrere and Alexandre, 2015; Li et al., 2016; Bennett et al., 2019). For example, the model proposed by Li et al. (2016) faces the problem of why it is less probable, for a memory deriving from a probabilistic pairing between the CS and US, to be extinguished, compared to a memory built through a full pairing of the CS and US. Their network, composed of four computational units representing LA, CeM, IL, and ITC, elegantly reproduces the phenomenon but has a weak relation to the underlying biological substrate, both at the level of single neurons and of the amygdala/mPFC network.

Another example of top-down model, which is the closest to our model for its aim to reproduce several experimental results (23 target experiments), is the one reported in Krasne et al. (2011). This model is built on a biologically grounded network comprising amygdala, cortex, and hippocampus, plus a second network composed of “theoretical units,” such as “reinforcing,” “extinction,” and “secondary reinforcing” units. Compared to this model, the model presented here reproduces a similar number of experimental datasets (25; see Supplementary Table 6) but it also presents some complementary features and novelties. The model presented here reproduces sixteen experiments not reproduced in the model presented by Krasne et al. (2011). With respect to the type of experiments reproduced by Krasne et al. (2011), the model presented here has a more focused target, in particular it does not consider hippocampus and contextual conditioning but performs a deeper study of conditioning, extinction, and reinstatement and the detailed mechanisms internal to the amygdala and the PL/IL underlying them. This stronger focus allowed the construction of a model architecture strictly constrained by empirical evidence. The few additional hypotheses that were introduced to allow the model to function and cover some relevant knowledge gaps in the literature were coherent with indirect empirical evidence or made predictions that are either supported by the literature or could be tested in future experiments.

Finally, we discuss here a study of Vlachos et al. (2011) that deals with the mechanism of emergence of fear and extinction neurons, simulating it through both an abstract firing-rate model and a biologically plausible spiking neuron model. Three differences with our model can be detected: (a) the model takes into account only the BA; (b) extinction is modeled with an experimental paradigm consisting in the CS exposure in a context that is different from the one used for conditioning (the contexts overlap up to 50%); (c) fear and extinction neurons are represented as two mutually inhibiting populations. In their model, fear and extinction neurons represent BA populations recruited in different contexts. In our paradigm fear and extinction neurons could be viewed as populations activated by the same context: extinction neurons (in particular in BAp3) are activated through a supplementary circuit comprising IL. Extinction neurons inhibit fear neurons through interneurons, as in Vlachos et al. (2011), but they are located outside the BA, in particular within the ITC. Moreover, in our model fear neurons do not suppress extinction neurons after fear re-establishment. Instead, this occurs because of the depotentiation of the connection between BA and IL, as described in the fourth hypothesis.

None of the top-down models include in the explanation of extinction the fundamental action of endocannabinoids and of DSI/E, with the exception of the models presented by Anastasio (2013) and Bennett et al. (2019) that however accounted for few target experiments.

### 4.3. Limitations of the Model and Future Work

Notwithstanding the strengths highlighted in the previous section, the model presented here has limitations that should be tackled in future work. First, we hypothesized that BAp5 is a persistent unit capable of undergoing a transient DSE but not a permanent between-session extinction. This is in line with the experiments of Kamprath et al. (2011) where fear extinction lasts for three sessions. However, protocols involving a larger number of sessions have shown a complete between-session extinction (Lebrón et al., 2004; Mao et al., 2013; An et al., 2017). In this respect, it has been shown that many repeated sessions of extinction involve a second mechanism that is independent of the IL and that rules out the inhibitory circuit driven by extinction neurons (Lebrón et al., 2004; An et al., 2017). One possibility is that this second mechanism is based on the depotentiation of the synaptic input from the CS-pathway to the fear circuit (Lin et al., 2003; Kim et al., 2007; Dalton et al., 2008; Mao et al., 2013). However, it is difficult to reconcile this depotentiation, which would result in the elimination of the memory trace in the amygdala, with the fact that fear can be easily reinstated (Maren and Holmes, 2016). It has been suggested that a prolonged extinction would result in a protection from spontaneous recovery of fear (Mao et al., 2013), but additional work would be required to incorporate this mechanism in a biologically grounded computational model. Another limitation of the model is that it does not consider neuromodulators. Different neuromodulators are released during fear memory formation and extinction and are involved in the modulation of amygdala and the mPFC functioning (Feenstra et al., 2001; Bissière et al., 2003; Hu et al., 2007; Hugues et al., 2007; Tully et al., 2007; Jiang et al., 2016; Salgado et al., 2016; Uematsu et al., 2017). For example, it has been found that dopamine might be relevant to support the US representation and learning processes within LA (Yu et al., 2017). Moreover, the interaction of different neuromodulators influences synaptic plasticity in complex ways that are still widely debated (Pawlak et al., 2010; Foncelle et al., 2018). Our aim was here to account for as many experiments as possible by introducing as few empirically-unsupported hypotheses as possible, so we addressed experiments where neuromodulators were not directly manipulated and had not an evident major role. Future work should thus address experiments involving neuromodulators to investigate how these might modulate the functioning of the circuit identified here, in particular by introducing additional hypotheses filling in the several knowledge gaps in the literature (e.g., see Carrere and Alexandre, 2015; Oliva et al., 2018).

Third, in our model we focused on a single mechanism of relapse, i.e., fear reinstatement, leaving out spontaneous recovery and renewal. We did this because spontaneous recovery and renewal require brain regions and protocols going beyond those considered here. Indeed, spontaneous recovery is strongly influenced by the timing of the exposure to fear of the used extinction protocol and by reconsolidation processes (Maren and Chang, 2006; Ponnusamy et al., 2016). Concerning renewal, this process strongly depends on the hippocampus (Ji and Maren, 2005; Zelikowsky et al., 2012). Given that our model does not includes the hippocampus as well as reconsolidation and context dependence, we decided to focus on fear reinstatement only.

The procedure of parameter search described in section 2.6 posed some limitations in both the number of brain regions included in the model and in the construction of its connectivity. Regarding the first issue, further increasing the complexity of the system by adding more brain areas or factors would make the manual parameter search extremely challenging. Concerning the second issue, testing different combinations of connectivity schemes would require, for each one, a parameter search and a following sensitivity analysis. In order to account for these issues is necessary an automatic methodology of parameter fitting, namely a genetic algorithm (Hojjatinia et al., 2020) or a brute-force search (Oliva et al., 2018). This different methodological approach could be the argument for a future work, allowing to analyze more complex behaviors, such as fear renewal (Ji and Maren, 2005; Zelikowsky et al., 2012) or freezing/flight behavior (Fadok et al., 2017).

A last limitation of the model, also shared with other computational models reviewed in the previous section and the target experiments themselves, is that the model identifies a complex evidence-based brain network underlying fear conditioning but it does not fully clarify its adaptive functions. Future work could thus aim to better understand the possible adaptive role of such mechanisms, or alternatively to reveal their non-adaptive, accidental nature (Gould and Lewontin, 1979). A possible approach to do so could be to embed the model within an embodied agent interacting with the environment (e.g., see Mannella et al., 2008, 2010; Balkenius et al., 2009) and to challenge it with several different tasks and conditions requiring fear expression/extinction/reinstatement so as to identify the possible adaptive role of the mechanisms studied with the model. These functions could then be tested in ecological empirical experiments.

## 5. Conclusions

The brain amygdala-prefrontal cortex system is at the core of classical conditioning, extinction, and reinstatement processes whose malfunctioning cause severe psychiatric disorders in humans. Years of animal investigations conducted with this paradigm have produced a vast amount of empirical results that need to be integrated in a unified view as they are expressed by the same brain system. The aim of this research was hence to propose a computational model that succeeded to reproduce many of those results (those reported in Table 1) on the basis of neural mechanisms grounded on empirical evidence and as few additional hypotheses as possible. In this respect, the model represents a possible explanation of how the several target phenomena might be produced by the same mechanisms, a result going beyond what was done by existing models.

Four specific hypotheses encompassed by the model contributed to its capacity to account for the target experiments: the existence of a LA-CeL circuit supporting the role of CeL as a break of freezing expression; an articulated proposal of how the endocannabinoid system might affect the role of the IL in extinction; the possible circuit connecting LA and CeM through BA, allowing the explanation of within- and between-session extinction; and the organization of the BA-PL-IL circuit explaining how the experience of a US alone could cause a reinstatement of the fear behavior.

There are additional issues that the model should address in future work, such as some aspects of plasticity, the role of neuromodulators, and the possible adaptive function of some of the proposed neural mechanisms. However, notwithstanding these limitations the model allows the explanation of a large number of target experiments, through evidence-based assumptions and few additional hypotheses, thus going beyond existing models.

## Data Availability Statement

The original contributions presented in the study are included in the article/Supplementary Material, further inquiries can be directed to the corresponding author/s.

## Author Contributions

AM, MP, and GB conceived the model, analyzed the results, and reviewed the paper. AM conducted the simulation. AM and GB wrote the paper. All authors contributed to the article and approved the submitted version.

## Conflict of Interest

The authors declare that the research was conducted in the absence of any commercial or financial relationships that could be construed as a potential conflict of interest.
