# Supplementary material for: A Computational Model Integrating Multiple Phenomena on Cued Fear Conditioning, Extinction, and Reinstatement
Source: Front Syst Neurosci. 2020 Sep 29;14:569108. doi: 10.3389/fnsys.2020.569108 (PMC7550679; doi:10.3389/fnsys.2020.569108)
Supplement: Supplementary file 1 [file Data_Sheet_1.PDF]

Supplementary Material

1 SUPPLEMENTARY FIGURES

Figure S1. Interactive model simulator software. The interface of the interactive software used for the manual search of the parameters. The system was controlled by some buttons to apply CS and US. Below are shown some of the sliding bars used to change the synaptic weights of the connections. This live system allowed to modify the parameters and to immediately observe the effect on the behavior of the model

Figure S2. Neural units activated/inactivated by a CS presentation after fear conditioning. Differently from figure 2, where the width of the lines is proportional to the initial connection weights, here all the fixed connections are represented with the same width. Instead, plastic connections are thicker/thinner in case of potentiation/depotentiation with respect to their initial weight. Connections and units in light-gray represent those that are inactive for the model inhibitions or lack of the US. The CS-LAp1, LAp2-CeL-ON and PLp-BAp4 connections are potentiated. This allows the CS to induce the CeL-OFF inactivation and the activation of the fear neurons in BA, even without the US.

Figure S3. Neural units activated/inactivated by a CS presentation after fear extinction, at the beginning of the second session. Differently from figure 2, where the width of the lines is proportional to the initial connection weights, here all the fixed connections are represented with the same width. Instead, plastic connections are thicker/thinner in case of potentiation/depotentiation with respect to their initial weight. Connections and units in light-gray are those that are inactive for the model inhibitions or lack of the US. While the CS-LAp1 (Clem and Huganir, 2010; Kim and Cho, 2017) and the CeL-ON connections remain potentiated, fear extinction drives the depotentiation of PLp-BAp4 (Vouimba and Maroun, 2011; Cho et al., 2013) connection and the potentiation of BAp2-ILp (Vouimba and Maroun, 2011) and BAp3-ITC (Amano et al., 2010) connections. These synaptic changes result in the CS-induced activation of ITC, that shutdown the fear unit BAp4.

Figure S4. Neural units activated/inactivated by a CS presentation after fear reinstatement. Differently from figure 2, where the width of the lines is proportional to the initial connection weights, here all the fixed connections are represented with the same width. Instead, plastic connections are thicker/thinner in case of potentiation/depotentiation with respect to their initial weight. Connections and units in light-gray are those that are inactive for the model inhibitions or lack of the US. During reinstatement there is a partial reversion of the plastic changes observed after extinction. In particular, PLp-BAp4 is potentiated and BAp2-ILp is depotentiated (Vouimba and Maroun, 2011). The model predicts that the depotentiation of the BAp3-ITC connection is not necessary to obtain fear reinstatement.

Figure S5. Neural units activated/inactivated during conditioning, when the US is substituted by the activation of the excitatory units in LA. Behavior of the system when fear conditioning is induced as described in Johansen et al. (2010). Differently from figure 2, where the width of the lines is proportional to the initial connection weights, here all the fixed connections are represented with the same width. Instead, plastic connections are thicker/thinner in case of potentiation/depotentiation with respect to their initial weight. Connections and units in light-gray are those that are inactive for the model inhibitions or lack of the US. Note that the extinction pathway (IL-BAp3-ITC) is activated together with the fear pathway. This reduces the activity of the fear unit BAp4 and, thus, the connection PLp-BAp4 is not potentiated.

Figure S6. IL is not necessary for the within-session extinction. The picture shows the effect of a CS presentation on the activation/inactivation of the neural units at the end of the first session of extinction, if the IL is inactivated as in Quirk et al. (2000). Differently from figure 2, where the width of the lines is proportional to the initial connection weights, here all the fixed connections are represented with the same width. Instead, plastic connections are thicker/thinner in case of potentiation/depotentiation with respect to their initial weight. Connections and units in light-gray are those that are inactive for the model inhibitions or lack of the US. Even though IL cortex is inactivated, DSE sets off the input from BA to CeM, carrying out the within-session extinction. However, if IL is not active, the connections BAp2-ILp and BAp3-ITC are not potentiated, and the connection PLp-BAp4 is not depotentiated, as should happen during fear extinction (Kim and Cho, 2017; Vouimba and Maroun, 2011; Cho et al., 2013; Amano et al., 2010).

Figure S7. Results of the sensitivity analysis targeting the synaptic weight parameters of the model. The shown lower and upper values of each parameter indicates the first value (in percent of the original level, explored in steps of $\pm 5\%$) that causes the failure of the reproduction of at least one target experiment. Some parameter ranges are truncated: values at -100% and $+100\%$ indicate that at those values the model still reproduces all experiments. In particular, some parameters (weights of connections ILp-BAp3, ILp-BAp4, ITC-BAp4, ILp-ITC, BAp3-ITC, LAvip-LApv, US-LAvip, US-ILu, US-CeM, ILp-BApv2) can span values much higher than $+100\%$ (see text). A value of -100% indicates that the synapse was set to zero; that is, it could be removed altogether without affecting the results.

Figure S8. Sensitivity analysis performed on the plasticity parameters. The chart shows the range of variation of the parameters of synaptic plasticity up to -100% and +100%. Many parameters (DSE BAp5-CeM, DSE BAp4-CeM, LTP CS-LAp1, DSI BAcck-BAp2, LTP BAp2-ILp, LTP BAp3-ITC, LTP LAp2-CeL-ON, LTP PLp-BAp4) span over a value much higher than these values (see text).

2 SUPPLEMENTARY TABLES

Table S1. Leaky units parameters. Each value represents the mean of the values reported in the corresponding references. Firing threshold was calculated by subtracting from the actual threshold the resting potential of the neurons.

Unit	τ (ms)	References	Threshold	References	ϕ (Hz)	References
LAp1, LAp2	20.6	Rosenkranz (2011); Fink and LeDoux (2018)	28.1	Faber and Sah (2004); Sosulina et al. (2010); Perkowski and Murphy (2011); Fink and LeDoux (2018)	35.5	Kaneko et al. (2008); Fink and LeDoux (2018)
LAvip, CeL-ON	13.3	Kaneko et al. (2008); Rhomberg et al. (2018)	27.2	Kaneko et al. (2008); Sosulina et al. (2010)	44.7	Kaneko et al. (2008); Sosulina et al. (2010); Rhomberg et al. (2018)
LApv, BApv, BAcck, CeL-OFF	13.0	Kaneko et al. (2008); Rhomberg et al. (2018)	31.9	Kaneko et al. (2008); Sosulina et al. (2010); Rhomberg et al. (2018)	144	Kaneko et al. (2008)
BAp1, BAp3, BAp4, BAp5	31	Kaneko et al. (2008)	28.1	Faber and Sah (2004); Sosulina et al. (2010); Perkowski and Murphy (2011); Fink and LeDoux (2018)	35.5	Kaneko et al. (2008); Fink and LeDoux (2018)
BAp2	15	Ehrlich et al. (2012)	28.1	Faber and Sah (2004); Sosulina et al. (2010); Perkowski and Murphy (2011); Fink and LeDoux (2018)	35.5	Kaneko et al. (2008); Fink and LeDoux (2018)
PLp, ILp	19	Povysheva et al. (2005)	22.2	Hedrich et al. (2014); Tai et al. (2014)	45.5	Chen et al. (2015)
PLs, ILs	29.2	Ali and Thomson (2007)	26.3	Hu and Agmon (2015)	75.5	Chen et al. (2015)

PLpv, ILpv	7.7	Povysheva et al. (2005)	30.8	Povysheva et al. (2005)	163.3	Chen et al. (2015)
ITC	23.5	Geracitano et al. (2007); Mańko et al. (2011)	41.2	Geracitano et al. (2007); Kaneko et al. (2008); Sosulina et al. (2010); Mańko et al. (2011); Gungor et al. (2015); Rhomberg et al. (2018)	30.4	Geracitano et al. (2007); Busti et al. (2011); Mańko et al. (2011)
CeM	35.6	Martina et al. (1999); Busti et al. (2011); Gungor et al. (2015)	27.5	Martina et al. (1999); Gungor et al. (2015)	25	Busti et al. (2011)

Table S2. Fixed connections of the model. List of connections, literature supporting their existence, and values of their synaptic weights found through the procedures illustrated in Section 2.6 of the main text. These values were used to produce all the simulations in the figures from 3 to 12.

Connection	References	Weight
US to LAp1	Romanski et al. (1993); Blair et al. (2001)	1.52
US to LAvip	Wolff et al. (2014); Krabbe et al. (2018); Rhomberg et al. (2018)	1.8
US to ILpv	Hypothesis	7.0
CS to LAp1	Romanski et al. (1993); Blair et al. (2001)	0.35
CS to LAp2	Blair et al. (2001)	1.0
CS to LApv	Wolff et al. (2014); Krabbe et al. (2018)	0.505
LAvip to LApv	Wolff et al. (2014); Krabbe et al. (2018); Rhomberg et al. (2018)	1.805
LAp1 to CeL-ON	Pape and Pare (2010); Li et al. (2013)	0.8
LAp2 to CeL-ON	Pape and Pare (2010); Li et al. (2013)	0.8
CeL-ON to CeL-OFF	Haubensak et al. (2010)	0.7
CeL-OFF to CeM	Haubensak et al. (2010)	2.265
LApv to LAp1	Wolff et al. (2014); Krabbe et al. (2018)	0.33
LApv to LAp2	Wolff et al. (2014); Krabbe et al. (2018)	0.4
LAp1 to BAp1	Stefanacci et al. (1992); Pitkänen et al. (1995); Savander et al. (1997)	7.0804

LAp1 to BAp2	Stefanacci et al. (1992); Pitkänen et al. (1995); Savander et al. (1997)	5.0
LAp1 to BAp5	Stefanacci et al. (1992); Pitkänen et al. (1995); Savander et al. (1997)	3.0
LAp1 to BAcck	Stefanacci et al. (1992); Pitkänen et al. (1995); Savander et al. (1997)	1.9
BAcck to BAp1	Vogel et al. (2016)	0.7
BAcck to BAp2	Vogel et al. (2016)	0.7
BAp1 to PLs	Courtin et al. (2014); Cummings and Clem (2020)	1.2
BAp1 to PLpv	McGarry and Carter (2016)	1.4
BAp1 to PLp	Senn et al. (2014)	2.2
BAp2 to ILs	Courtin et al. (2014); Cummings and Clem (2020)	2.5
BAp2 to ILpv	McGarry and Carter (2016)	0.3
BAp2 to ILp	Senn et al. (2014)	3.3
PLs to PLpv	Courtin et al. (2014); Cummings and Clem (2020)	1.35
PLpv to PLp	Courtin et al. (2014)	1.2
PLp to ILp	Marek et al. (2018)	1.2
PLp to BAp4	Vertes (2004); Cho et al. (2013); Courtin et al. (2014)	3.6
PLp to BApv	Cho et al. (2013)	0.8
ILs to ILpv	Courtin et al. (2014); Cummings and Clem (2020)	0.5
ILpv to ILp	Courtin et al. (2014)	1.5
ILp to ITC	Vertes (2004); Pinard et al. (2012); Cho et al. (2013)	1.0
ILp to BAp3	Vertes (2004); Cho et al. (2013); Courtin et al. (2014)	3.3
BApv to BAp4	Cho et al. (2013)	0.6
BAp3 to ITC	Amano et al. (2010)	1.0
ITC to BAp4	Asede et al. (2015)	8.0
BAp4 to CeM	Asede et al. (2015)	2.0
BAp5 to CeM	Asede et al. (2015)	1.8

Table S3. Plastic connections. List of the model plastic connections and type of plasticity and plasticity parameters used to produce all the simulations in the figures from 3 to 12.

**Connection**	**Plasticity type**	η	σ
CS-pathway to LAp1	LTP	0.00012	0.85
LAp2 to CeL-ON	LTP	0.0002	0.85

PLp to BAp4	LTP	0.001	0.5
PLp to BAp4	LTD	0.00000014	0.5
BAp2 to IL	LTP	0.0000002	0.5
BAp2 to IL	LTD	0.0001	0.5
BAp3 to ITC	LTP	0.000000018	0.5
BAcck to BAp2	DSI	0.0000005	-
BAp4 to CeM	DSE	0.000004	-
BAp5 to CeM	DSE	0.000005	-

Table S4. Constraints used in the sensitivity analysis. List of constraints and references supporting their existence.

<table>
<tr><th>Constraints</th><th>References</th></tr>
<tr><td>After conditioning, CeM must fire more than 70% of its maximum activity following CS.</td><td rowspan="2">Pape and Pare (2010); Amano et al. (2010).</td></tr>
<tr><td>After extinction CeM must fire less than 20% of its maximum following CS.</td></tr>
<tr><td>After conditioning, the fear unit BAp4 must fire more than 20% of its maximum following CS.</td><td>Herry et al. (2008).</td></tr>
<tr><td>After conditioning the persistent unit BAp1 must fire more than 20% of its maximum following CS.</td><td rowspan="4">Amano et al. (2011); Trouche et al. (2013).</td></tr>
<tr><td>After extinction the persistent unit BAp1 must fire more than 20% of its maximum following CS.</td></tr>
<tr><td>After conditioning the persistent unit BAp5 must fire more than 20% of its maximum following CS.</td></tr>
<tr><td>After extinction the persistent unit BAp5 must fire more than 20% of its maximum following CS.</td></tr>
<tr><td>After conditioning the extinction unit BAp3 must fire less than 20% of its maximum following CS.</td><td rowspan="2">Herry et al. (2008).</td></tr>
<tr><td>After extinction the extinction unit BAp3 must fire more than 20% of its maximum following CS.</td></tr>
<tr><td>In the last extinction trial the unit BAp2 that project to ILp must fire more than it did in the first trial.</td><td>Senn et al. (2014).</td></tr>
<tr><td>After reinstatement CeM must fire more than 70% of its maximum following CS.</td><td>Rescorla and Heth (1975).</td></tr>
<tr><td>After reinstatement ILp must fire less than 20% of its maximum following CS.</td><td>Hitora-Imamura et al. (2015).</td></tr>
<tr><td>After conditioning LAp1 stimulation from CS-pathway must exert a PSP at least higher than 20%.</td><td>McKernan and Shinnick-Gallagher (1997); Tsvetkov et al. (2002); Schafe et al. (2005).</td></tr>
<tr><td>After conditioning CeL-ON stimulation from LAp2 must exert a PSP at least higher than 20%.</td><td>Li et al. (2013).</td></tr>
</table>

After extinction ITC stimulation from BAp3 must exert a PSP at least higher than 20%.	Amano et al. (2010).
After conditioning BAp4 stimulation from PLp must exert a PSP at least higher than 20%.	Vouimba and Maroun (2011).
After extinction BAp4 stimulation from PLp must exert a PSP at least lower than 20%.	
After extinction ILp stimulation from BAp2 must exert a PSP must exert a PSP at least higher than 20%.	
After reinstatement ILp stimulation from BAp2 must exert a PSP at least lower than 20%.	
After reinstatement BAp4 stimulation from PLp must exert a PSP must exert a PSP at least higher than 20%.	
The PL inactivation should not influence conditioning, but only fear expression: during the third trial of conditioning CeM activation should be lower than 50% if the PL is inactivated, but during the test trial, when the PL is reactivated, should be at least 90% than control, as in the target experiment.	Corcoran and Quirk (2007).
As in the target experiment, potentiation of CS-pathway to LA should not be sufficient to exert conditioning. If the weight of this connection is increased of 300%, CS-induced CeM activation should be lower than 30%. After conditioning, CeM activation should be higher than 70%, but should return to pre-conditioning level if CS-pathway to LAp1 weight is decreased to baseline level.	Nabavi et al. (2014).
LA inactivation should impair conditioning: CeM activation following CS should be lower than 50% compared to control, as in the target experiment.	Wilensky et al. (2006).
CeL inactivation should impair conditioning: CeM activation following CS should be lower than 50% compared to control, as in the target experiment	Wilensky et al. (2006).
Before conditioning CeL-ON must fire less than 20% of its maximum following CS.	Ciocchi et al. (2010).
After conditioning CeL-ON must fire more than 70% of its maximum following CS.	
Before conditioning CeL-OFF must fire more than 70% of its maximum following CS.	
After conditioning CeL-OFF must fire less than 20% of its maximum following CS.	

As in the target experiment, substituting US with LAp1 and LAp2 maximal activation during conditioning should induce CeM to be conditioned to CS, although its activity should be substantially lower than classical conditioning (between 50% and 60% of maximal activity).	Johansen et al. (2010).
Between-session extinction: during the first trial of the second extinction session CeM must fire less than during the first trial of the first extinction session.	Quirk et al. (2000); Kamprath et al. (2006); Plendl and Wotjak (2010).
Blocking DSI/E in whole amygdala should impairs between- and within-session extinction. The ratio between CeM activation in control and in DSI/E impaired system during the last two trials of session 1 and the last two trials of session 2 should be lower than respectively 0.34, 0.4, 0.46, 0.41, mirroring the measurement of freezing during the last two trials of sessions 1 and 3 of the target experiment.	Marsicano et al. (2002).
Blocking DSE in CeM in the first session should impairs within-but not between-session extinction. CeM activity during the first two and the last two trials of the second extinction session should be no more than 5% different between controls and impaired system. The ratio between CeM activation in control and in DSI/E impaired system during the last two trials of session 1 should be less than respectively 0.21, 0.33.	Kamprath et al. (2011).
Blocking DSE in BA in the first session should impairs between-but not within-session extinction. CeM activity during the last two trials of the first and second extinction session should be no more than 5% different between controls and impaired system. The ratio between CeM activation in control and in DSI/E impaired system during the first three trials of session 2 should be less than 0.47, mirroring the average of the measurement of freezing in the first three trials of session 2 in the target experiment.	Kamprath et al. (2011).

Inactivation of the IL does not influence conditioning or within-session extinction, but only between-session extinction. The last trial of conditioning, the last two trials of the first and of the second extinction session should be no more than 5% different between controls and impaired system. The ratio between CeM activation in control and in the IL impaired system during the first three trials of session 2 should be less than 0.27, mirroring the average of the measurement of freezing in the first three trials of session 2 in the target experiment	Quirk et al. (2000).
As in the target experiment, activation of the IL should speed up extinction: given that in our control extinction takes 7 trials, we defined extinction to occur earlier if during trials 3 and 5 in the IL stimulated system CeM activity is equal or less than, respectively, trials 5 and 7 of the control.	Vidal-Gonzalez et al. (2006).
As in the target experiment, activation of ILp projecting PLp connection should speed up extinction: given that in our control extinction takes 7 trials, we defined extinction to occur earlier if during trials 3 and 5 in the IL stimulated system CeM activity is equal or less than, respectively, trials 5 and 7 of the control.	Marek et al. (2018).
As in the target experiment, deactivation of ILp projecting PLp connection should slow down extinction: given that in our control extinction takes 7 trials, we defined extinction to occur later if during trials 9 and 11 in impaired system CeM activity is equal or higher than, respectively, trials 7 and 9 of the control.	Marek et al. (2018).

Table S5. Experimental targets of the model

**Experimental target**	**References**
**Basic conditioning, extinction, and reinstatement processes**	
During fear conditioning and extinction three classes of neurons emerge: fear neurons in BA and CeM, extinction neurons in BA, ITC, and the IL, and persistent neurons in LA and BA.	Repa et al. (2001); Milad and Quirk (2002); Santini et al. (2008); Herry et al. (2008); Amano et al. (2010); An et al. (2012).
BA neurons that project to the IL are progressively recruited during within-session extinction.	Senn et al. (2014).

An US presented after extinction reinstates the freezing response to the CS.	Rescorla and Heth (1975).
Fear reinstatement is associated with a reduced activity in the IL.	Hitora-Imamura et al. (2015).
**Synaptic plasticity**	
Fear conditioning potentiates the synapses between the CS-pathway and LA pyramidal neurons.	Rogan and LeDoux (1995); Rogan et al. (1997); McKernan and Shinnick-Gallagher (1997); Tsvetkov et al. (2002); Schafe et al. (2005).
Fear conditioning potentiates the excitatory synapses between LA and CeL neurons.	Li et al. (2013).
Fear conditioning strengthens the connections between mPFC and BA.	Vouimba and Maroun (2011).
Fear extinction does not eliminates LTP of the CS-pathway established during conditioning.	Kim and Cho (2017).
Fear extinction strengthens the connections between BA and the mPFC.	Vouimba and Maroun (2011).
Fear extinction weakens the connections between the mPFC and BA.	Vouimba and Maroun (2011); Cho et al. (2013).
Fear extinction induces LTP at the synapses between BA and ITC.	Amano et al. (2010).
Fear reinstatement reverses changes induced by fear extinction in BA-mPFC and in mPFC-BA connections.	Vouimba and Maroun (2011).
**Fear conditioning**	
Inhibition of the PL reduces fear expression, but does not influence conditioning.	Corcoran and Quirk (2007).

LA is necessary for fear conditioning expression: once conditioning is established, it can be eliminated (as in reinstatement) through the optogenetic induction of LTD and LTP at the synapses between the CS-pathway and LA pyramidal neurons.	Nabavi et al. (2014); Kim and Cho (2017).
Fear conditioning cannot be established solely by optogenetic induction of LTP at the synapses between CS-pathway and LA pyramidal neurons.	Nabavi et al. (2014).
CeL is necessary for fear conditioning.	Wilensky et al. (2006); Ciocchi et al. (2010).
Two classes of CeL neurons respond differently to CS after fear conditioning, one by increasing its firing and the other one by decreasing its firing rate.	Ciocchi et al. (2010).
Fear conditioning can be obtained without the US by pairing the CS with the optogenetic depolarization of pyramidal neurons in LA.	Johansen et al. (2010).
**Fear extinction**	
Blockade of endocannabinoids in the whole amygdala impaires within- and between-session extinction.	Marsicano et al. (2002); Kamprath et al. (2011); Plendl and Wotjak (2010).
Blockade of endocannabinoids in CeM reduces within-session extinction, but spares between-session extinction.	Kamprath et al. (2011).
Blockade of endocannabinoids in BA impairs between-session extinction, but spares within-session extinction.	Kamprath et al. (2011).
The IL inhibition does not influence fear expression, conditioning, or within-session extinction, but impairs between-session extinction.	Quirk et al. (2000); Do-Monte et al. (2015); Kim et al. (2016b); Bloodgood et al. (2018), but see Lebrón et al. (2004).
The IL stimulation speeds up fear extinction.	Vidal-Gonzalez et al. (2006); Adhikari et al. (2015).

Stimulation of the PL projections to the IL speeds up fear extinction.	Marek et al. (2018).
Inhibition of the PL projections to the IL impairs early extinction.	Marek et al. (2018).

Table S6. List of target experiments on fear extinction, and models discussed in the paper that addressed them. 'This model' refers to the model reported in this paper.

Experimental target and references	Models
Cued fear conditioning: Quirk et al. (2000); Quirk and Mueller (2008).	**This model**; MorÉn (2001); Burgos and Murillo-Rodríguez (2007); Li et al. (2009); Vlachos et al. (2011); Krasne et al. (2011); John et al. (2013); Kim et al. (2013b); Moustafa et al. (2013); Carrere and Alexandre (2015); Kim et al. (2016a); Feng et al. (2016); Li et al. (2016); Bennett et al. (2019).
Cued fear conditioning requires CS onset preceding US onset: Ayres et al. (1987); Albert and Ayres (1997); Esmorís-Arranz et al. (2003).	Krasne et al. (2011).
Cued fear conditioning requires plasticity in LA: Rodrigues et al. (2001); Sotres-Bayon et al. (2007).	**This model**; Krasne et al. (2011).
Fear conditioning potentiates the synapses between the CS-pathway and LA pyramidal neurons: McKernan and Shinnick-Gallagher (1997); Tsvetkov et al. (2002); Schafe et al. (2005).	**This model**; Li et al. (2009); John et al. (2013); Kim et al. (2013b); Li et al. (2016).
Fear conditioning potentiates the excitatory synapses between LA and CeL neurons: Li et al. (2013).	**This model**.

Fear conditioning strengthens the connections between the mPFC and BA: Vouimba and Maroun (2011).	**This model**.
Once conditioning is established, it can be erasered and reinstated through the optogenetic induction of LTD and LTP at the synapses between the CS-pathway and LA pyramidal neurons: Nabavi et al. (2014); Kim and Cho (2017).	**This model**.
Fear conditioning cannot be established solely by optogenetic induction of LTP at the synapses between CS-pathway and LA pyramidal neurons: Nabavi et al. (2014).	**This model**.
Cued fear conditioning should be also expressed outside the context where conditioning occurred: Bouton and King (1983).	Krasne et al. (2011).
Second-order fear conditioning: Marlin (1983); Helmstetter and Fanselow (1989).	Krasne et al. (2011).
Immediate shock deficit: Fanselow (1990).	Krasne et al. (2011).
CeL is necessary for fear conditioning: Wilensky et al. (2006); Ciocchi et al. (2010).	**This model**.
Two classes of CeL neurons respond differently to CS after fear conditioning, one increasing and the other decreasing its firing: Ciocchi et al. (2010).	**This model**; Carrere and Alexandre (2015).
Pre-conditioning ablation of the hippocampus allows contextual fear conditioning, but accentuate the immediate shock deficit: Wiltgen et al. (2006).	Krasne et al. (2011).
Pre-conditioning ablation of the hippocampus makes contextual fear conditioning dependent on the IL: Zelikowsky et al. (2010).	Krasne et al. (2011).
An already conditioned CS blocks the conditioning to a new stimulus if presented with it: McNish et al. (2000); Cole and McNally (2007).	MorÉn (2001); Krasne et al. (2011).
Conditioned response decrease if CS is delivered in a different context than conditioning: Gordon et al. (1981); Riccio et al. (1984); Millin and Riccio (2004).	Burgos and Murillo-Rodríguez (2007).
Post- but not pre-training ablation of BA impairs conditioning: Anglada-Figueroa and Quirk (2005); Jimenez and Maren (2009).	Krasne et al. (2011).
Contextual fear conditioning depends on a functional BA: Helmstetter (1992); Fanselow and Kim (1994); Calandreau et al. (2005).	Krasne et al. (2011).

Hippocampus impairment blocks contextual fear conditioning: Young et al. (1994); Stiedl et al. (2000); Bast et al. (2003); Quinn et al. (2005); Parsons and Otto (2008); Schenberg and Oliveira (2008); Raineki et al. (2010).	Krasne et al. (2011).
Hippocampus impairment does not affect contextual fear conditioning if the context is long-familiar: Young et al. (1994); Anagnostaras et al. (2001).	Krasne et al. (2011) .
Hippocampus impairment does not affect cued fear conditioning: Kim and Fanselow (1992).	Krasne et al. (2011); Moustafa et al. (2013).
Recent but not remote contextual fear is erased by hippocampus ablation: Kim and Fanselow (1992).	Krasne et al. (2011).
mPFC is essential for the expression of remote but not recent trace fear conditioning: Quinn et al. (2008).	Krasne et al. (2011).
Inhibition of the PL reduces fear expression, but does not influence conditioning: Corcoran and Quirk (2007).	**This model**.
During cued fear conditioning in LA appear transiently plastic cells: Repa et al. (2001).	Kim et al. (2013b).
Acetilcholine controls cued vs. contextual fear conditioning: Calandreau et al. (2006).	Carrere and Alexandre (2015).
During fear conditioning, neurons in LA with higher excitability are more likely to be incorporated in the fear engram: Han et al. (2007, 2009).	Kim et al. (2013a, 2016a); Feng et al. (2016).
The size of the fear engram in LA is regulated by synaptic inhibition: Morrison et al. (2016).	Feng et al. (2016).
Fear conditioning can be obtained without the US by pairing the CS with the optogenetic depolarization of pyramidal neurons in LA: Johansen et al. (2010).	**This model**.

Fear extinction: Quirk et al. (2000); Quirk and Mueller (2008).	**This model**; MorÉn (2001); Burgos and Murillo-Rodríguez (2007); Li et al. (2009); Vlachos et al. (2011); Krasne et al. (2011); John et al. (2013); Kim et al. (2013b); Moustafa et al. (2013); Carrere and Alexandre (2015); Li et al. (2016); Bennett et al. (2019).
Extinction is impaired by periacqueductal opiate receptors antagonists: McNally et al. (2004); Parsons et al. (2010).	Krasne et al. (2011).
Extinction requires synaptic plasticity in BA: Sotres-Bayon et al. (2007); Zimmerman and Maren (2010) (but see Tronson et al. (2006)).	**This model**; Krasne et al. (2011).
Extinction does not require synaptic plasticity in BA: Tronson et al. (2006) (but see Sotres-Bayon et al. (2007); Zimmerman and Maren (2010)).	Moustafa et al. (2013).
Fear extinction strengthens the connections between BA and the mPFC: Vouimba and Maroun (2011).	**This model**.
Fear extinction weakens the connections between the mPFC and BA: Vouimba and Maroun (2011); Cho et al. (2013).	**This model**.
Fear extinction induces LTP at the synapses between BA and ITC: Amano et al. (2010).	**This model**; Li et al. (2011); John et al. (2013).
Fear extinction does not eliminates LTP of the CS-pathway established during conditioning: Kim and Cho (2017).	**This model**; John et al. (2013); Li et al. (2016).

BA neurons that project to the IL are progressively recruited during within-session extinction: Senn et al. (2014).	**This model**.
The IL units increase their activity during extinction recall: Milad and Quirk (2002).	**This model**; Moustafa et al. (2013); Bennett et al. (2019).
The IL inhibition does not influence fear expression, conditioning, or within-session extinction, but impairs between-session extinction: Quirk et al. (2000); Bloodgood et al. (2018); Do-Monte et al. (2015); Kim et al. (2016b) (but see Lebrón et al. (2004)).	**This model**.
The IL inhibition impairs late phase of the first session extinction: Lebrón et al. (2004).	Moustafa et al. (2013).
The IL is not necessary for extinction recall: Do-Monte et al. (2015) (but see Laurent and Westbrook (2009); Kim et al. (2016b)).	Li et al. (2016).
The IL stimulation speeds up fear extinction: Vidal-Gonzalez et al. (2006); Adhikari et al. (2015).	**This model**.
Stimulation of the PL projections to the IL speeds up fear extinction: Marek et al. (2018).	**This model**.
Inhibition of the PL projections to the IL impairs early extinction: Marek et al. (2018).	**This model**.
If hippocampus is removed before conditioning, cued fear extinction becomes context specific: Wilson et al. (1995); Frohardt et al. (2000); Zelikowsky et al. (2012) (but see Ji and Maren (2005)).	Krasne et al. (2011); Moustafa et al. (2013).
Extinction can not be acquired if acetylcholine is depleted: Prado-Alcalá et al. (1994).	Carrere and Alexandre (2015).
Partial reinforcement extinction effect: Leonard (1975); Rescorla (1999).	Li et al. (2016).
Blockade of endocannabinoids in the whole amygdala impaires within-session extinction: Marsicano et al. (2002); Kamprath et al. (2011); Plendl and Wotjak (2010).	**This model**; Anastasio (2013); Bennett et al. (2019).
Blockade of endocannabinoids in the whole amygdala impaires between-session extinction: Marsicano et al. (2002); Kamprath et al. (2011); Plendl and Wotjak (2010).	**This model**.
Blockade of endocannabinoids in CeM reduces within-session extinction, but spares between-session extinction: Kamprath et al. (2011).	**This model**.

If endocannabinoids are blocked in BA there is an impairment of between- but not within-session extinction: Kamprath et al. (2011).	**This model**.
Emergence of fear neurons: Herry et al. (2008); Amano et al. (2010).	**This model**; Li et al. (2009); Vlachos et al. (2011); Carrere and Alexandre (2015); Li et al. (2016); Bennett et al. (2019).
Emergence of extinction neurons: Milad and Quirk (2002); Herry et al. (2008); Santini et al. (2008); Amano et al. (2010).	**This model**; Li et al. (2009); Vlachos et al. (2011); Carrere and Alexandre (2015); Li et al. (2016); Bennett et al. (2019).
Emergence of persistent neurons: Repa et al. (2001); Amano et al. (2011); An et al. (2012); Trouche et al. (2013).	**This model**; Vlachos et al. (2011); Kim et al. (2013b); Li et al. (2016).
GABAergic neurons are essential for extinction: Harris and Westbrook (1998); Chhatwal et al. (2005).	Li et al. (2009), **This model**.
NMDA receptors are required for extinction: Santini et al. (2001); Suzuki et al. (2004); Sotres-Bayon et al. (2007).	Li et al. (2009).
Fear reinstatement: Rescorla and Heth (1975); Maren and Holmes (2016).	**This model**.
Fear reinstatement is associated with a reduced activity in the IL: Hitora-Imamura et al. (2015).	**This model**.
Fear reinstatement reverses changes induced by fear extinction in mPFC to BA and in BA to the mPFC connections: Vouimba and Maroun (2011).	**This model**.

Fear renewal: Maren and Holmes (2016).	Burgos and Murillo-Rodríguez (2007); Krasne et al. (2011); Vlachos et al. (2011); Carrere and Alexandre (2015).
Spontaneous recovery of fear: Maren and Holmes (2016).	Li et al. (2009).

REFERENCES

Adhikari, A., Lerner, T. N., Finkelstein, J., Pak, S., Jennings, J. H., Davidson, T. J., et al. (2015). Basomedial amygdala mediates top-down control of anxiety and fear. *Nature* 527, 179

Albert, M. and Ayres, J. J. (1997). One-trial simultaneous and backward excitatory fear conditioning in rats: Lick suppression, freezing, and rearing to cs compounds and their elements. *Animal Learning & Behavior* 25, 210–220

Ali, A. B. and Thomson, A. M. (2007). Synaptic $\alpha 5$ subunit–containing gabaa receptors mediate ipsps elicited by dendrite-preferring cells in rat neocortex. *Cerebral cortex* 18, 1260–1271

Amano, T., Duvarci, S., Popa, D., and Paré, D. (2011). The fear circuit revisited: contributions of the basal amygdala nuclei to conditioned fear. *Journal of Neuroscience* 31, 15481–15489

Amano, T., Unal, C. T., and Paré, D. (2010). Synaptic correlates of fear extinction in the amygdala. *Nature neuroscience* 13, 489

An, B., Hong, I., and Choi, S. (2012). Long-term neural correlates of reversible fear learning in the lateral amygdala. *Journal of Neuroscience* 32, 16845–16856

Anagnostaras, S. G., Gale, G. D., and Fanselow, M. S. (2001). Hippocampus and contextual fear conditioning: recent controversies and advances. *Hippocampus* 11, 8–17

Anastasio, T. (2013). Computational search for hypotheses concerning the endocannabinoid contribution to the extinction of fear conditioning. *Frontiers in computational neuroscience* 7, 74

Anglada-Figueroa, D. and Quirk, G. J. (2005). Lesions of the basal amygdala block expression of conditioned fear but not extinction. *Journal of Neuroscience* 25, 9680–9685

Asede, D., Bosch, D., Lüthi, A., Ferraguti, F., and Ehrlich, I. (2015). Sensory inputs to intercalated cells provide fear-learning modulated inhibition to the basolateral amygdala. *Neuron* 86, 541–554

Ayres, J. J., Haddad, C., and Albert, M. (1987). One-trial excitatory backward conditioning as assessed by conditioned suppression of licking in rats: Concurrent observations of lick suppression and defensive behaviors. *Animal Learning & Behavior* 15, 212–217

Bast, T., Zhang, W.-N., and Feldon, J. (2003). Dorsal hippocampus and classical fear conditioning to tone and context in rats: Effects of local nmda-receptor blockade and stimulation. *Hippocampus* 13, 657–675

Bennett, M. R., Farnell, L., Gibson, W. G., and Lagopoulos, J. (2019). A model of amygdala function following plastic changes at specific synapses during extinction. *Neurobiology of stress* 10, 100159

Blair, H. T., Schafe, G. E., Bauer, E. P., Rodrigues, S. M., and LeDoux, J. E. (2001). Synaptic plasticity in the lateral amygdala: a cellular hypothesis of fear conditioning. *Learning & memory* 8, 229–242

Bloodgood, D. W., Sugam, J. A., Holmes, A., and Kash, T. L. (2018). Fear extinction requires infralimbic cortex projections to the basolateral amygdala. *Translational psychiatry* 8, 60

Bouton, M. E. and King, D. A. (1983). Contextual control of the extinction of conditioned fear: tests for the associative value of the context. *Journal of Experimental Psychology: Animal Behavior Processes* 9, 248

Burgos, J. E. and Murillo-Rodríguez, E. (2007). Neural-network simulations of two context-dependence phenomena. *Behavioural processes* 75, 242–249

Busti, D., Geracitano, R., Whittle, N., Dalezios, Y., Mańko, M., Kaufmann, W., et al. (2011). Different fear states engage distinct networks within the intercalated cell clusters of the amygdala. *Journal of Neuroscience* 31, 5131–5144

Calandreau, L., Desmedt, A., Decorte, L., and Jaffard, R. (2005). A different recruitment of the lateral and basolateral amygdala promotes contextual or elemental conditioned association in pavlovian fear conditioning. *Learning & Memory* 12, 383–388

Calandreau, L., Trifilieff, P., Mons, N., Costes, L., Marien, M., Marighetto, A., et al. (2006). Extracellular hippocampal acetylcholine level controls amygdala function and promotes adaptive conditioned emotional response. *Journal of Neuroscience* 26, 13556–13566

Carrere, M. and Alexandre, F. (2015). A pavlovian model of the amygdala and its influence within the medial temporal lobe. *Frontiers in systems neuroscience* 9, 41

Chen, I.-W., Helmchen, F., and Lütcke, H. (2015). Specific early and late oddball-evoked responses in excitatory and inhibitory neurons of mouse auditory cortex. *Journal of Neuroscience* 35, 12560–12573

Chhatwal, J. P., Myers, K. M., Ressler, K. J., and Davis, M. (2005). Regulation of gephyrin and gabaa receptor binding within the amygdala after fear acquisition and extinction. *Journal of Neuroscience* 25, 502–506

Cho, J.-H., Deisseroth, K., and Bolshakov, V. Y. (2013). Synaptic encoding of fear extinction in mpfc-amygdala circuits. *Neuron* 80, 1491–1507

Ciocchi, S., Herry, C., Grenier, F., Wolff, S. B., Letzkus, J. J., Vlachos, I., et al. (2010). Encoding of conditioned fear in central amygdala inhibitory circuits. *Nature* 468, 277

Clem, R. L. and Huganir, R. L. (2010). Calcium-permeable ampa receptor dynamics mediate fear memory erasure. *science* 330, 1108–1112

Cole, S. and McNally, G. P. (2007). Opioid receptors mediate direct predictive fear learning: Evidence from one-trial blocking. *Learning & Memory* 14, 229–235

Corcoran, K. A. and Quirk, G. J. (2007). Activity in prelimbic cortex is necessary for the expression of learned, but not innate, fears. *Journal of Neuroscience* 27, 840–844

Courtin, J., Chaudun, F., Rozeske, R. R., Karalis, N., Gonzalez-Campo, C., Wurtz, H., et al. (2014). Prefrontal parvalbumin interneurons shape neuronal activity to drive fear expression. *Nature* 505, 92

Cummings, K. A. and Clem, R. L. (2020). Prefrontal somatostatin interneurons encode fear memory. *Nature neuroscience* 23, 61–74

Do-Monte, F. H., Manzano-Nieves, G., Quiñones-Laracuente, K., Ramos-Medina, L., and Quirk, G. J. (2015). Revisiting the role of infralimbic cortex in fear extinction with optogenetics. *Journal of Neuroscience* 35, 3607–3615

Ehrlich, D., Ryan, S., and Rainnie, D. (2012). Postnatal development of electrophysiological properties of principal neurons in the rat basolateral amygdala. *The Journal of physiology* 590, 4819–4838

Esmorís-Arranz, F. J., Pardo-Vázquez, J. L., and Vázquez-García, G. A. (2003). Differential effects of forward or simultaneous conditioned stimulus-unconditioned stimulus intervals on the defensive behavior system of the norway rat (rattus norvegicus). *Journal of Experimental Psychology: Animal*

Behavior Processes 29, 334

Faber, E. L. and Sah, P. (2004). Opioids inhibit lateral amygdala pyramidal neurons by enhancing a dendritic potassium current. *Journal of Neuroscience* 24, 3031–3039

Fanselow, M. S. (1990). Factors governing one-trial contextual conditioning. *Animal Learning & Behavior* 18, 264–270

Fanselow, M. S. and Kim, J. J. (1994). Acquisition of contextual pavlovian fear conditioning is blocked by application of an nmda receptor antagonist d, l-2-amino-5-phosphonovaleric acid to the basolateral amygdala. *Behavioral neuroscience* 108, 210

Feng, F., Samarth, P., Paré, D., and Nair, S. S. (2016). Mechanisms underlying the formation of the amygdalar fear memory trace: a computational perspective. *Neuroscience* 322, 370–376

Fink, A. E. and LeDoux, J. E. (2018). β-adrenergic enhancement of neuronal excitability in the lateral amygdala is developmentally gated. *Journal of neurophysiology* 119, 1658–1664

Frohardt, R. J., Guarraci, F. A., and Bouton, M. E. (2000). The effects of neurotoxic hippocampal lesions on two effects of context after fear extinction. *Behavioral neuroscience* 114, 227

Geracitano, R., Kaufmann, W. A., Szabo, G., Ferraguti, F., and Capogna, M. (2007). Synaptic heterogeneity between mouse paracapsular intercalated neurons of the amygdala. *The Journal of physiology* 585, 117–134

Gordon, W. C., McCracken, K. M., Dess-Beech, N., and Mowrer, R. R. (1981). Mechanisms for the cueing phenomenon: The addition of the cueing context to the training memory. *Learning and Motivation* 12, 196–211

Gungor, N. Z., Yamamoto, R., and Paré, D. (2015). Optogenetic study of the projections from the bed nucleus of the stria terminalis to the central amygdala. *Journal of neurophysiology* 114, 2903–2911

Han, J.-H., Kushner, S. A., Yiu, A. P., Cole, C. J., Matynia, A., Brown, R. A., et al. (2007). Neuronal competition and selection during memory formation. *science* 316, 457–460

Han, J.-H., Kushner, S. A., Yiu, A. P., Hsiang, H.-L. L., Buch, T., Waisman, A., et al. (2009). Selective erasure of a fear memory. *Science* 323, 1492–1496

Harris, J. A. and Westbrook, R. F. (1998). Evidence that gaba transmission mediates context-specific extinction of learned fear. *Psychopharmacology* 140, 105–115

Haubensak, W., Kunwar, P. S., Cai, H., Ciocchi, S., Wall, N. R., Ponnusamy, R., et al. (2010). Genetic dissection of an amygdala microcircuit that gates conditioned fear. *Nature* 468, 270

Hedrich, U. B., Liautard, C., Kirschenbaum, D., Pofahl, M., Lavigne, J., Liu, Y., et al. (2014). Impaired action potential initiation in gabaergic interneurons causes hyperexcitable networks in an epileptic mouse model carrying a human nav1. 1 mutation. *Journal of Neuroscience* 34, 14874–14889

Helmstetter, F. J. (1992). Contribution of the amygdala to learning and performance of conditional fear. *Physiology & Behavior* 51, 1271–1276

Helmstetter, F. J. and Fanselow, M. S. (1989). Differential second-order aversive conditioning using contextual stimuli. *Animal Learning & Behavior* 17, 205–212

Herry, C., Ciocchi, S., Senn, V., Demmou, L., Müller, C., and Lüthi, A. (2008). Switching on and off fear by distinct neuronal circuits. *Nature* 454, 600

Hitora-Imamura, N., Miura, Y., Teshirogi, C., Ikegaya, Y., Matsuki, N., and Nomura, H. (2015). Prefrontal dopamine regulates fear reinstatement through the downregulation of extinction circuits. *Elife* 4, e08274

Hu, H. and Agmon, A. (2015). Properties of precise firing synchrony between synaptically coupled cortical interneurons depend on their mode of coupling. *Journal of neurophysiology* 114, 624–637

Ji, J. and Maren, S. (2005). Electrolytic lesions of the dorsal hippocampus disrupt renewal of conditional fear after extinction. *Learning & Memory* 12, 270–276

Jimenez, S. A. and Maren, S. (2009). Nuclear disconnection within the amygdala reveals a direct pathway to fear. *Learning & memory* 16, 766–768

Johansen, J. P., Hamanaka, H., Monfils, M. H., Behnia, R., Deisseroth, K., Blair, H. T., et al. (2010). Optical activation of lateral amygdala pyramidal cells instructs associative fear learning. *Proceedings of the National Academy of Sciences* 107, 12692–12697

John, Y. J., Bullock, D., Zikopoulos, B., and Barbas, H. (2013). Anatomy and computational modeling of networks underlying cognitive-emotional interaction. *Frontiers in human neuroscience* 7, 101

Kamprath, K., Marsicano, G., Tang, J., Monory, K., Bisogno, T., Di Marzo, V., et al. (2006). Cannabinoid cb1 receptor mediates fear extinction via habituation-like processes. *Journal of Neuroscience* 26, 6677–6686

Kamprath, K., Romo-Parra, H., Häring, M., Gaburro, S., Doengi, M., Lutz, B., et al. (2011). Short-term adaptation of conditioned fear responses through endocannabinoid signaling in the central amygdala. *Neuropsychopharmacology* 36, 652

Kaneko, K., Tamamaki, N., Owada, H., Kakizaki, T., Kume, N., Totsuka, M., et al. (2008). Noradrenergic excitation of a subpopulation of gabaergic cells in the basolateral amygdala via both activation of nonselective cationic conductance and suppression of resting k+ conductance: A study using glutamate decarboxylase 67–green fluorescent protein knock-in mice. *Neuroscience* 157, 781–797

Kim, D., Pare, D., and Nair, S. S. (2013a). Assignment of model amygdala neurons to the fear memory trace depends on competitive synaptic interactions. *Journal of Neuroscience* 33, 14354–14358

Kim, D., Paré, D., and Nair, S. S. (2013b). Mechanisms contributing to the induction and storage of pavlovian fear memories in the lateral amygdala. *Learning & Memory* 20, 421–430

Kim, D., Samarth, P., Feng, F., Pare, D., and Nair, S. S. (2016a). Synaptic competition in the lateral amygdala and the stimulus specificity of conditioned fear: a biophysical modeling study. *Brain Structure and Function* 221, 2163–2182

Kim, H.-S., Cho, H.-Y., Augustine, G. J., and Han, J.-H. (2016b). Selective control of fear expression by optogenetic manipulation of infralimbic cortex after extinction. *Neuropsychopharmacology* 41, 1261

Kim, J. J. and Fanselow, M. S. (1992). Modality-specific retrograde amnesia of fear. *Science* 256, 675–677

Kim, W. B. and Cho, J.-H. (2017). Encoding of discriminative fear memory by input-specific ltp in the amygdala. *Neuron* 95, 1129–1146

Krabbe, S., Gründemann, J., and Lüthi, A. (2018). Amygdala inhibitory circuits regulate associative fear conditioning. *Biological psychiatry* 83, 800–809

Krasne, F. B., Fanselow, M., and Zelikowsky, M. (2011). Design of a neurally plausible model of fear learning. *Frontiers in behavioral neuroscience* 5, 41

Laurent, V. and Westbrook, R. F. (2009). Inactivation of the infralimbic but not the prelimbic cortex impairs consolidation and retrieval of fear extinction. *Learning & memory* 16, 520–529

Lebrón, K., Milad, M. R., and Quirk, G. J. (2004). Delayed recall of fear extinction in rats with lesions of ventral medial prefrontal cortex. *Learning & memory* 11, 544–548

Leonard, D. W. (1975). Partial reinforcement effects in classical aversive conditioning in rabbits and human beings. *Journal of comparative and physiological psychology* 88, 596

Li, G., Amano, T., Pare, D., and Nair, S. S. (2011). Impact of infralimbic inputs on intercalated amygdala neurons: a biophysical modeling study. *Learning & Memory* 18, 226–240

Li, G., Nair, S. S., and Quirk, G. J. (2009). A biologically realistic network model of acquisition and extinction of conditioned fear associations in lateral amygdala neurons. *Journal of neurophysiology* 101, 1629–1646

Li, H., Penzo, M. A., Taniguchi, H., Kopec, C. D., Huang, Z. J., and Li, B. (2013). Experience-dependent modification of a central amygdala fear circuit. *Nature neuroscience* 16, 332

Li, Y., Nakae, K., Ishii, S., and Naoki, H. (2016). Uncertainty-dependent extinction of fear memory in an amygdala-mpfc neural circuit model. *PLoS computational biology* 12, e1005099

Mańko, M., Geracitano, R., and Capogna, M. (2011). Functional connectivity of the main intercalated nucleus of the mouse amygdala. *The Journal of physiology* 589, 1911–1925

Marek, R., Xu, L., Sullivan, R. K., and Sah, P. (2018). Excitatory connections between the prelimbic and infralimbic medial prefrontal cortex show a role for the prelimbic cortex in fear extinction. *Nature neuroscience* 21, 654

Maren, S. and Holmes, A. (2016). Stress and fear extinction. *Neuropsychopharmacology* 41, 58

Marlin, N. A. (1983). Second-order conditioning using a contextual stimulus as s 1. *Animal Learning & Behavior* 11, 290–294

Marsicano, G., Wotjak, C. T., Azad, S. C., Bisogno, T., Rammes, G., Cascio, M. G., et al. (2002). The endogenous cannabinoid system controls extinction of aversive memories. *Nature* 418, 530

Martina, M., Royer, S., and Paré, D. (1999). Physiological properties of central medial and central lateral amygdala neurons. *Journal of neurophysiology* 82, 1843–1854

McGarry, L. M. and Carter, A. G. (2016). Inhibitory gating of basolateral amygdala inputs to the prefrontal cortex. *Journal of Neuroscience* 36, 9391–9406

McKernan, M. and Shinnick-Gallagher, P. (1997). Fear conditioning induces a lasting potentiation of synaptic currents in vitro. *Nature* 390, 607

McNally, G. P., Pigg, M., and Weidemann, G. (2004). Opioid receptors in the midbrain periaqueductal gray regulate extinction of pavlovian fear conditioning. *Journal of Neuroscience* 24, 6912–6919

McNish, K. A., Gewirtz, J. C., and Davis, M. (2000). Disruption of contextual freezing, but not contextual blocking of fear-potentiated startle, after lesions of the dorsal hippocampus. *Behavioral Neuroscience* 114, 64

Milad, M. R. and Quirk, G. J. (2002). Neurons in medial prefrontal cortex signal memory for fear extinction. *Nature* 420, 70

Millin, P. M. and Riccio, D. C. (2004). Is the context shift effect a case of retrieval failure? the effects of retrieval enhancing treatments on forgetting under altered stimulus conditions in rats. *Journal of Experimental Psychology: Animal Behavior Processes* 30, 325

MorÉn, J., Christian Balkenius (2001). Emotional learning: A computational model of the amygdala. *Cybernetics & Systems* 32, 611–636

Morrison, D. J., Rashid, A. J., Yiu, A. P., Yan, C., Frankland, P. W., and Josselyn, S. A. (2016). Parvalbumin interneurons constrain the size of the lateral amygdala engram. *Neurobiology of learning and memory* 135, 91–99

Moustafa, A. A., Gilbertson, M. W., Orr, S. P., Herzallah, M. M., Servatius, R. J., and Myers, C. E. (2013). A model of amygdala–hippocampal–prefrontal interaction in fear conditioning and extinction in animals. *Brain and cognition* 81, 29–43

Nabavi, S., Fox, R., Proulx, C. D., Lin, J. Y., Tsien, R. Y., and Malinow, R. (2014). Engineering a memory with ltd and ltp. *Nature* 511, 348

Pape, H.-C. and Pare, D. (2010). Plastic synaptic networks of the amygdala for the acquisition, expression, and extinction of conditioned fear. *Physiological reviews* 90, 419–463

Parsons, R., Gafford, G. M., and Helmstetter, F. J. (2010). Regulation of extinction-related plasticity by opioid receptors in the ventrolateral periaqueductal gray matter. *Frontiers in behavioral neuroscience* 4, 44

Parsons, T. C. and Otto, T. (2008). Temporary inactivation of dorsal hippocampus attenuates explicitly nonspatial, unimodal, contextual fear conditioning. *Neurobiology of learning and memory* 90, 261–268

Perkowski, J. J. and Murphy, G. G. (2011). Deletion of the mouse homolog of kcnab2, a gene linked to monosomy 1p36, results in associative memory impairments and amygdala hyperexcitability. *Journal of Neuroscience* 31, 46–54

Pinard, C. R., Mascagni, F., and McDonald, A. J. (2012). Medial prefrontal cortical innervation of the intercalated nuclear region of the amygdala. *Neuroscience* 205, 112–124

Pitkänen, A., Stefanacci, L., Farb, C. R., Go, G.-G., Ledoux, J. E., and Amaral, D. G. (1995). Intrinsic connections of the rat amygdaloid complex: projections originating in the lateral nucleus. *Journal of Comparative Neurology* 356, 288–310

Plendl, W. and Wotjak, C. T. (2010). Dissociation of within-and between-session extinction of conditioned fear. *Journal of Neuroscience* 30, 4990–4998

Povysheva, N., Gonzalez-Burgos, G., Zaitsev, A., Kröner, S., Barrionuevo, G., Lewis, D., et al. (2005). Properties of excitatory synaptic responses in fast-spiking interneurons and pyramidal cells from monkey and rat prefrontal cortex. *Cerebral Cortex* 16, 541–552

Prado-Alcalá, R. A., Haiek, M., Rivas, S., Roldan-Roldan, G., and Quirarte, G. L. (1994). Reversal of extinction by scopolamine. *Physiology & behavior* 56, 27–30

Quinn, J. J., Loya, F., Ma, Q. D., and Fanselow, M. S. (2005). Dorsal hippocampus nmda receptors differentially mediate trace and contextual fear conditioning. *Hippocampus* 15, 665–674

Quinn, J. J., Ma, Q. D., Tinsley, M. R., Koch, C., and Fanselow, M. S. (2008). Inverse temporal contributions of the dorsal hippocampus and medial prefrontal cortex to the expression of long-term fear memories. *Learning & memory* 15, 368–372

Quirk, G. J. and Mueller, D. (2008). Neural mechanisms of extinction learning and retrieval. *Neuropsychopharmacology* 33, 56

Quirk, G. J., Russo, G. K., Barron, J. L., and Lebron, K. (2000). The role of ventromedial prefrontal cortex in the recovery of extinguished fear. *Journal of Neuroscience* 20, 6225–6231

Raineki, C., Holman, P. J., Debiec, J., Bugg, M., Beasley, A., and Sullivan, R. M. (2010). Functional emergence of the hippocampus in context fear learning in infant rats. *Hippocampus* 20, 1037–1046

Repa, J. C., Muller, J., Apergis, J., Desrochers, T. M., Zhou, Y., and LeDoux, J. E. (2001). Two different lateral amygdala cell populations contribute to the initiation and storage of memory. *Nature neuroscience* 4, 724

Rescorla, R. A. (1999). Partial reinforcement reduces the associative change produced by nonreinforcement. *Journal of Experimental Psychology: Animal Behavior Processes* 25, 403

Rescorla, R. A. and Heth, C. D. (1975). Reinstatement of fear to an extinguished conditioned stimulus. *Journal of Experimental Psychology: Animal Behavior Processes* 1, 88

Rhomberg, T., Rovira-Esteban, L., Vikór, A., Paradiso, E., Kremser, C., Nagy-Pál, P., et al. (2018). Vasoactive intestinal polypeptide-immunoreactive interneurons within circuits of the mouse basolateral amygdala. *Journal of Neuroscience* 38, 6983–7003

Riccio, D. C., Richardson, R., and Ebner, D. L. (1984). Memory retrieval deficits based upon altered contextual cues: a paradox. *Psychological bulletin* 96, 152

Rodrigues, S. M., Schafe, G. E., and LeDoux, J. E. (2001). Intra-amygdala blockade of the nr2b subunit of the nmda receptor disrupts the acquisition but not the expression of fear conditioning. *Journal of Neuroscience* 21, 6889–6896

Rogan, M. T. and LeDoux, J. E. (1995). Ltp is accompanied by commensurate enhancement of auditory-evoked responses in a fear conditioning circuit. *Neuron* 15, 127–136

Rogan, M. T., Stäubli, U. V., and LeDoux, J. E. (1997). Fear conditioning induces associative long-term potentiation in the amygdala. *Nature* 390, 604

Romanski, L. M., Clugnet, M.-C., Bordi, F., and LeDoux, J. E. (1993). Somatosensory and auditory convergence in the lateral nucleus of the amygdala. *Behavioral neuroscience* 107, 444

Rosenkranz, J. A. (2011). Neuronal activity causes rapid changes of lateral amygdala neuronal membrane properties and reduction of synaptic integration and synaptic plasticity in vivo. *Journal of Neuroscience* 31, 6108–6120

Santini, E., Muller, R. U., and Quirk, G. J. (2001). Consolidation of extinction learning involves transfer from nmda-independent to nmda-dependent memory. *Journal of Neuroscience* 21, 9009–9017

Santini, E., Quirk, G. J., and Porter, J. T. (2008). Fear conditioning and extinction differentially modify the intrinsic excitability of infralimbic neurons. *Journal of Neuroscience* 28, 4028–4036

Savander, V., Miettinen, R., Ledoux, J., and Pitkänen, A. (1997). Lateral nucleus of the rat amygdala is reciprocally connected with basal and accessory basal nuclei: a light and electron microscopic study. *Neuroscience* 77, 767–781

Schafe, G. E., Doyère, V., and LeDoux, J. E. (2005). Tracking the fear engram: the lateral amygdala is an essential locus of fear memory storage. *Journal of Neuroscience* 25, 10010–10014

Schenberg, E. E. and Oliveira, M. G. M. (2008). Effects of pre or posttraining dorsal hippocampus d-ap5 injection on fear conditioning to tone, background, and foreground context. *Hippocampus* 18, 1089–1093

Senn, V., Wolff, S. B., Herry, C., Grenier, F., Ehrlich, I., Gründemann, J., et al. (2014). Long-range connectivity defines behavioral specificity of amygdala neurons. *Neuron* 81, 428–437

Sosulina, L., Graebenitz, S., and Pape, H.-C. (2010). Gabaergic interneurons in the mouse lateral amygdala: a classification study. *Journal of neurophysiology* 104, 617–626

Sotres-Bayon, F., Bush, D. E., and LeDoux, J. E. (2007). Acquisition of fear extinction requires activation of nr2b-containing nmda receptors in the lateral amygdala. *Neuropsychopharmacology* 32, 1929

Stefanacci, L., Farb, C. R., Pitkänen, A., Go, G., Ledoux, J. E., and Amaral, D. G. (1992). Projections from the lateral nucleus to the basal nucleus of the amygdala: a light and electron microscopic pha-l study in the rat. *Journal of Comparative Neurology* 323, 586–601

Stiedl, O., Birkenfeld, K., Palve, M., and Spiess, J. (2000). Impairment of conditioned contextual fear of c57bl/6j mice by intracerebral injections of the nmda receptor antagonist apv. *Behavioural brain research* 116, 157–168

Suzuki, A., Josselyn, S. A., Frankland, P. W., Masushige, S., Silva, A. J., and Kida, S. (2004). Memory reconsolidation and extinction have distinct temporal and biochemical signatures. *Journal of Neuroscience* 24, 4787–4795

Tai, C., Abe, Y., Westenbroek, R. E., Scheuer, T., and Catterall, W. A. (2014). Impaired excitability of somatostatin-and parvalbumin-expressing cortical interneurons in a mouse model of dravet syndrome. *Proceedings of the National Academy of Sciences* 111, E3139–E3148

Tronson, N. C., Wiseman, S. L., Olausson, P., and Taylor, J. R. (2006). Bidirectional behavioral plasticity of memory reconsolidation depends on amygdalar protein kinase a. *Nature neuroscience* 9, 167

Trouche, S., Sasaki, J. M., Tu, T., and Reijmers, L. G. (2013). Fear extinction causes target-specific remodeling of perisomatic inhibitory synapses. *Neuron* 80, 1054–1065

Tsvetkov, E., Carlezon Jr, W. A., Benes, F. M., Kandel, E. R., and Bolshakov, V. Y. (2002). Fear conditioning occludes ltp-induced presynaptic enhancement of synaptic transmission in the cortical pathway to the lateral amygdala. *Neuron* 34, 289–300

Vertes, R. P. (2004). Differential projections of the infralimbic and prelimbic cortex in the rat. *Synapse* 51, 32–58

Vidal-Gonzalez, I., Vidal-Gonzalez, B., Rauch, S. L., and Quirk, G. J. (2006). Microstimulation reveals opposing influences of prelimbic and infralimbic cortex on the expression of conditioned fear. *Learning & memory* 13, 728–733

Vlachos, I., Herry, C., Lüthi, A., Aertsen, A., and Kumar, A. (2011). Context-dependent encoding of fear and extinction memories in a large-scale network model of the basal amygdala. *PLoS computational biology* 7, e1001104

Vogel, E., Krabbe, S., Gründemann, J., Cusulin, J. I. W., and Lüthi, A. (2016). Projection-specific dynamic regulation of inhibition in amygdala micro-circuits. *Neuron* 91, 644–651

Vouimba, R.-M. and Maroun, M. (2011). Learning-induced changes in mpfc–bla connections after fear conditioning, extinction, and reinstatement of fear. *Neuropsychopharmacology* 36, 2276

Wilensky, A. E., Schafe, G. E., Kristensen, M. P., and LeDoux, J. E. (2006). Rethinking the fear circuit: the central nucleus of the amygdala is required for the acquisition, consolidation, and expression of pavlovian fear conditioning. *Journal of Neuroscience* 26, 12387–12396

Wilson, A., Brooks, D. C., and Bouton, M. E. (1995). The role of the rat hippocampal system in several effects of context in extinction. *Behavioral neuroscience* 109, 828

Wiltgen, B. J., Sanders, M. J., Anagnostaras, S. G., Sage, J. R., and Fanselow, M. S. (2006). Context fear learning in the absence of the hippocampus. *Journal of Neuroscience* 26, 5484–5491

Wolff, S. B., Gründemann, J., Tovote, P., Krabbe, S., Jacobson, G. A., Müller, C., et al. (2014). Amygdala interneuron subtypes control fear learning through disinhibition. *Nature* 509, 453

Young, S. L., Bohenek, D. L., and Fanselow, M. S. (1994). Nmda processes mediate anterograde amnesia of contextual fear conditioning induced by hippocampal damage: immunization against amnesia by context preexposure. *Behavioral neuroscience* 108, 19

Zelikowsky, M., Bissiere, S., Abdipranato, A., Hast, T., Vissel, B., and Fanselow, M. S. (2010). Contextual fear formed in the absence of the hippocampus is fleeting and requires infralimbic cortex. *Soc. Neurosci. Abstr.*

Zelikowsky, M., Pham, D. L., and Fanselow, M. S. (2012). Temporal factors control hippocampal contributions to fear renewal after extinction. *Hippocampus* 22, 1096–1106

Zimmerman, J. M. and Maren, S. (2010). Nmda receptor antagonism in the basolateral but not central amygdala blocks the extinction of pavlovian fear conditioning in rats. *European Journal of Neuroscience* 31, 1664–1670
