# Supplementary figures and images for: A Computational Model Integrating Multiple Phenomena on Cued Fear Conditioning, Extinction, and Reinstatement
Source: Front Syst Neurosci. 2020 Sep 29;14:569108. doi: 10.3389/fnsys.2020.569108 (PMC7550679; doi:10.3389/fnsys.2020.569108)

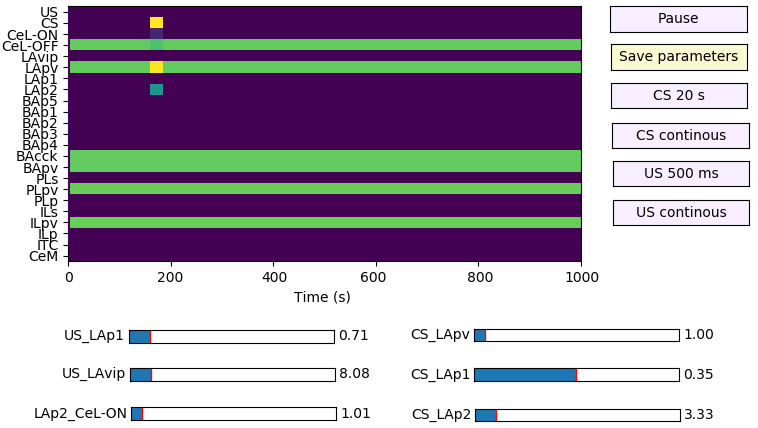

Supplement: Supplementary file 2 [file Image_1.TIF]

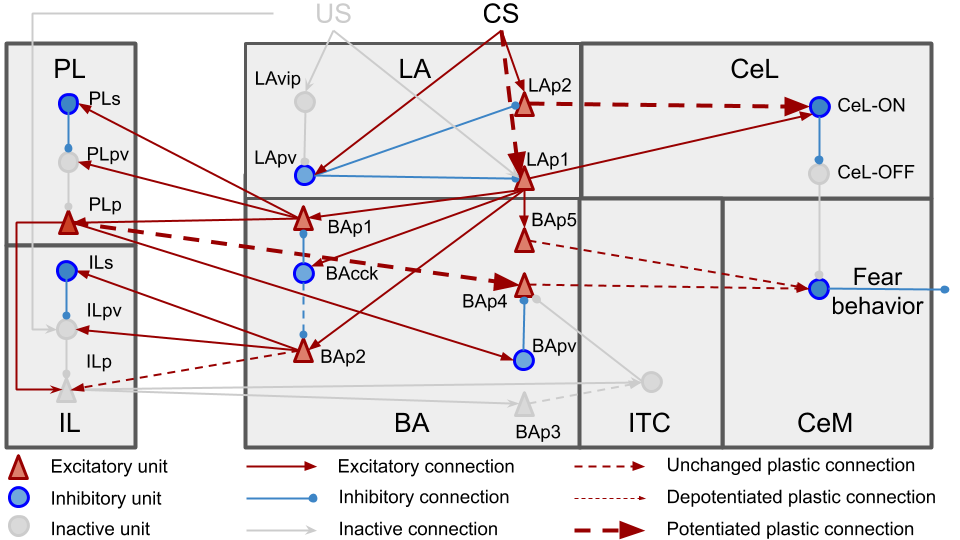

Supplement: Supplementary file 3 [file Image_2.TIF]

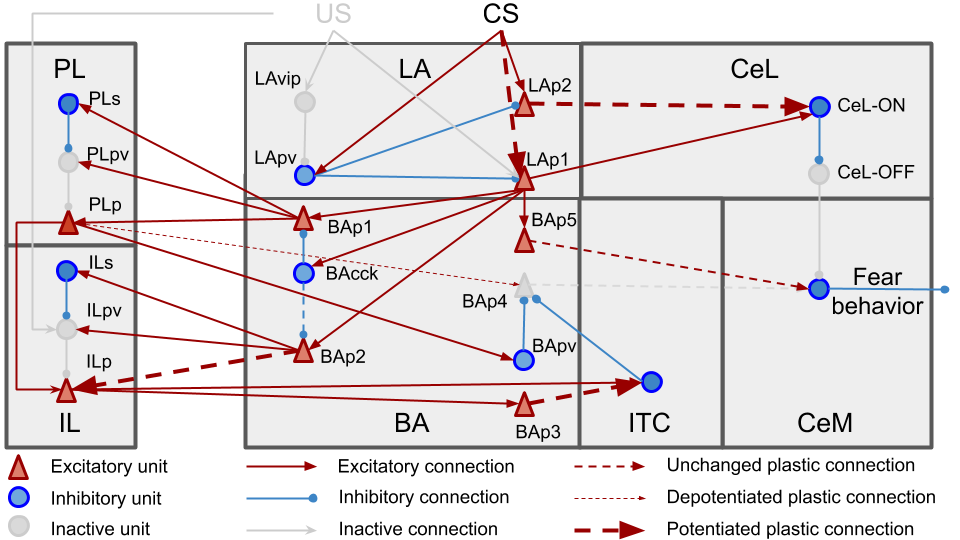

Supplement: Supplementary file 4 [file Image_3.TIF]

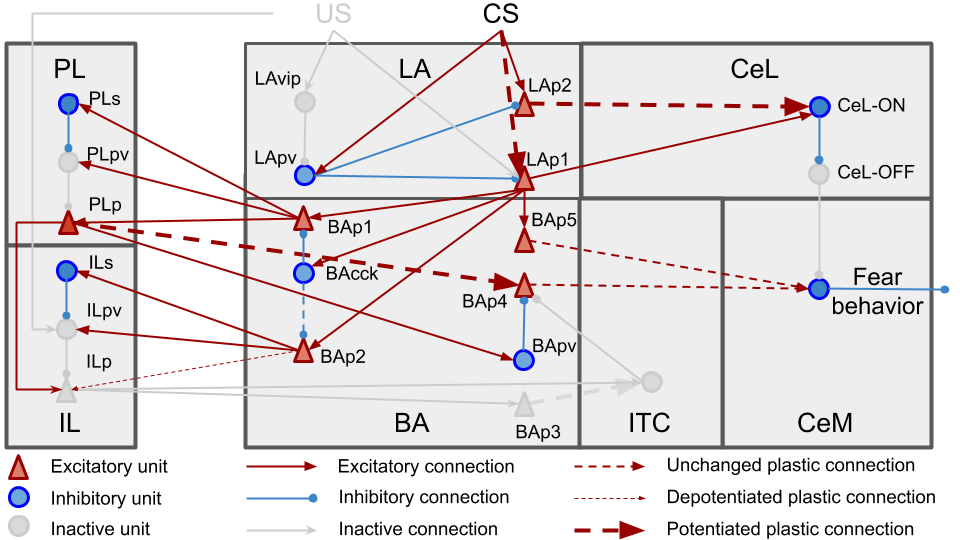

Supplement: Supplementary file 5 [file Image_4.TIF]

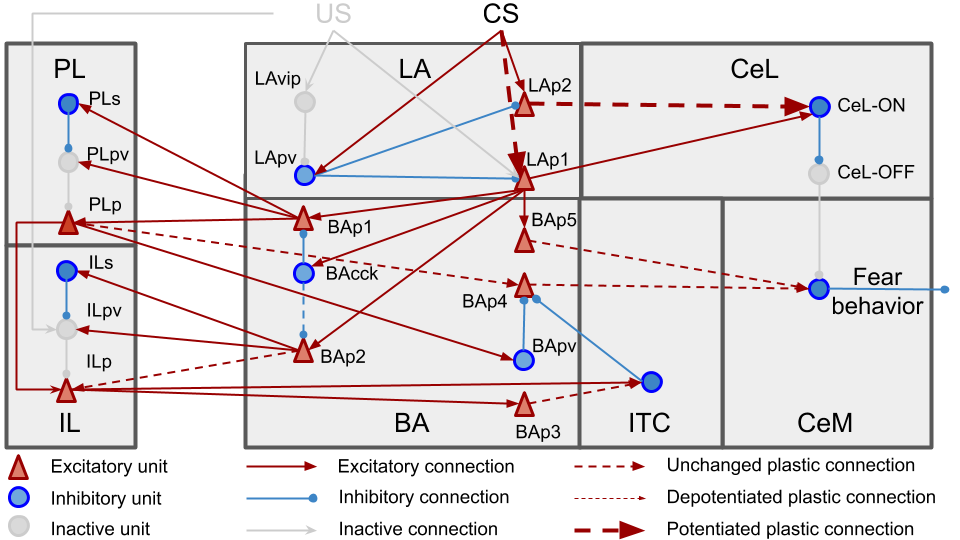

Supplement: Supplementary file 6 [file Image_5.TIF]

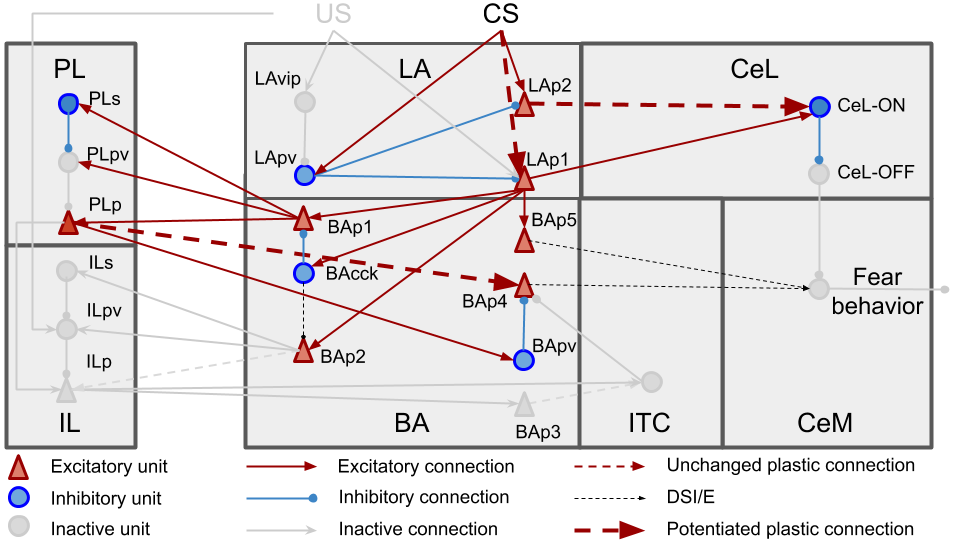

Supplement: Supplementary file 7 [file Image_6.TIF]

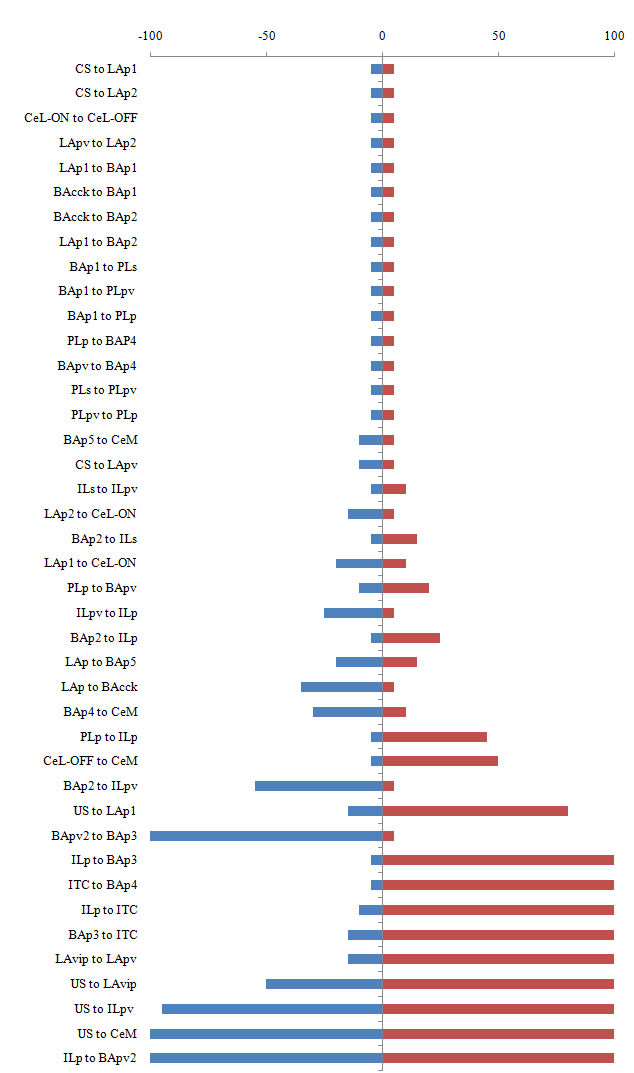

Supplement: Supplementary file 8 [file Image_7.TIF]

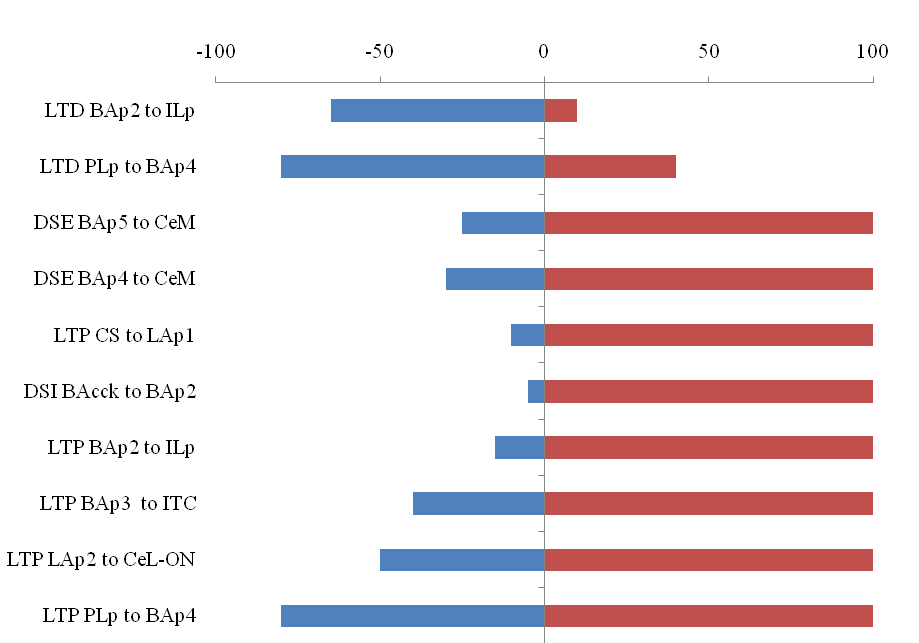

Supplement: Supplementary file 9 [file Image_8.tif]
